# Translating AI to the Bedside with Physician Buy-In: Recommendations from a Meta-Analysis and Systematic Review of the Literature

**DOI:** 10.3390/bioengineering12121363

**Published:** 2025-12-16

**Authors:** Grace Hwang, Seyyed Sina Hejazian, Alireza Vafaei Sadr, Jennifer K. Wagner, Pingxu Hao, Ajith Vemuri, Yuki Kawamura, Khalid Nawab, Shadi Hijjawi, Ramin Zand, Vida Abedi

**Affiliations:** 1 College of Medicine, Penn State University, Hershey, PA 17033, USA; 2 Department of Neurology, Penn State University College of Medicine, Hershey, PA 17033, USA; sina.hej95@gmail.com (S.S.H.); ramin.zand@gmail.com (R.Z.); 3 Department of Public Health Sciences, Penn State University College of Medicine, Hershey, PA 17033, USA; 4 School of Engineering Design and Innovation, Penn State University, University Park, PA 16827, USA; 5 Department of Anthropology, Penn State University, University Park, PA 16827, USA; 6 Department of Biomedical Engineering, Penn State University, University Park, PA 16827, USA; 7 Penn State Dickinson Law, University Park, PA 16827, USA; 8 Rock Ethics Institute, Penn State University, University Park, PA 16827, USA; 9 Institute for Computational and Data Sciences, Penn State University, University Park, PA 16827, USA; 10 Huck Institutes of the Life Sciences, Penn State University, University Park, PA 16827, USA; 11 School of Clinical Medicine, University of Cambridge, Cambridge CB2 0AH, UK; 12 Department of Medicine, Penn State University College of Medicine, Hershey, PA 17033, USAshijjawi@pennstatehealth.psu.edu (S.H.)

**Keywords:** artificial intelligence, physician buy-in, knowledge, attitude

## Abstract

Background: Artificial intelligence (AI) is increasingly being used in healthcare. Despite its promise, physicians and trainees remain cautiously optimistic. This systematic review and meta-analysis aimed to assess knowledge and attitudes toward AI and to provide recommendations for AI buy-in by physicians. Methods: Searches of PubMed-OVID-IEEE-Scopus, and Web-of-Science for studies in 2013–2024 identified 11,437 records. One-hundred-and-fifteen met inclusion criteria. Fifty-three studies reported quantitative data on physicians’/trainees’ knowledge and were included in the meta-analysis. Results: Our meta-analysis estimated that only 19.6% of physicians and trainees have high overall AI knowledge, while 36.3% have low knowledge. Fifty-five studies evaluated the depth of AI knowledge. These studies consistently concluded that most physicians or trainees possess only moderate conceptual knowledge of AI, and their technical knowledge is usually limited. Qualitative evaluations also highlighted that a high level of conceptual AI knowledge is associated with greater receptiveness to AI implementation in medicine. We identified five major barriers to translating AI to the bedside with physician buy-in. Conclusion: Although physicians and trainees are generally receptive to AI, many barriers hinder adoption. To address them, we recommend establishing standardized AI education and workforce training, involving clinicians early in AI design, clarifying legal and regulatory issues, leveraging insights from clinical decision support system implementation to reduce workflow challenges, and integrating patient-centered communication principles to enhance trust and transparency.

## 1. Introduction

Artificial intelligence (AI) and its subsets of machine learning (ML) and deep learning (DL) are being increasingly utilized as healthcare solutions for relieving physician cognitive load, replacing administrative tasks, triaging patients, and augmenting medical knowledge [1]. Despite the promising applications of AI, reception from physicians and physician-trainees has been cautiously optimistic. Research on the acceptance of AI by physicians and trainees has cited concerns about job replacement and workforce de-skilling. Additional concerns include legal uncertainty, erosion of the physician-patient relationship, algorithmic bias, and workflow integration and technical challenges [2,3].

Recent reviews on physician and trainee attitudes towards AI have identified barriers and opportunities for better engagement. For instance, Bhandari and colleagues (2021) [4] evaluated 14 quantitative studies on the attitudes of medical students, physicians, and patients toward AI in radiology. While students were concerned about job replacement and patients were concerned about malpractice accountability and lack of human interaction, clinicians were optimistic about AI as a diagnostic aid [4]. A systematic review of 19 studies surveying radiologists found that participants were excited about AI’s potential but reserved about its anticipated impact on radiology [5]. Additionally, in a review of 10 quantitative studies, scholars reported that medical students with various specialty interests had positive attitudes toward AI, but most students had low knowledge and limited AI skills [6]. The study highlighted the need for improved educational infrastructure to enhance AI competencies across various clinical specialties.

Although the use of AI applications in various clinical specialties is increasing rapidly [7,8,9,10], successful translations of AI from proof-of-concept to actual bedside implementation demand more than technological maturity; it critically relies on physician buy-in, which impacts tool adoption, workflow integration, trust, and sustainable use. Prior literature has examined attitudes toward AI within single specialties or trainee groups, but no review has synthesized these findings into actionable strategies for achieving clinician acceptance at the point of care. Given that AI systems ultimately fail without clinician trust, readiness, and willingness to use them, understanding the determinants of physician buy-in is essential for any AI intended for bedside deployment. Therefore, a comprehensive review of recent developments is warranted to better understand physicians’ knowledge, perceptions, and barriers regarding AI adoption. Based on our systematic review of the literature, we developed a set of five recommendations aimed at maximizing physician and trainee engagement in the implementation and bedside integration of AI-based tools across a broad range of specialties.

## 2. Materials and Methods

### 2.1. Information Sources and Search Strategy

This study was conducted and reported in accordance with the Preferred Reporting Items for Systematic Reviews and Meta-Analyses 2020 Checklist (PRISMA) [11]. The PROSPERO registration number for this systematic review is CRD420251244608. Search queries were conducted using PubMed, OVID, IEEE, Scopus, and Web of Science for any studies published between 1 January 2013, and 13 December 2024, without filters on study design, geography, or language. Given the fast-paced development of AI applications in healthcare, the search was restricted to the most recent 12 years. The following keywords and Medical Subject Headings terms were included in the search protocol: artificial intelligence, machine intelligence, machine learning, attitude, perspective, perception, acceptance, adoption, physician, clinician, and medical students.

### 2.2. Eligibility Criteria

We included studies with original, quantitative data on the knowledge, attitudes, or perspectives of current physicians or physician-trainees regarding AI in healthcare or specific AI clinical decision support systems (CDSS). We excluded studies that (1) did not discuss AI in healthcare; (2) did not evaluate knowledge, attitudes, or perspectives; (3) evaluated populations outside of physicians or physician-trainees; (4) were review articles or editorials; or (5) had inadequate or insufficient details on key data elements.

### 2.3. Data Extraction

After the removal of duplicate and irrelevant records, the screening was performed by GH and AS. Full-text reviews were completed by GH, AS, and PH to compile study methodology, participant characteristics, variables used to characterize attitudes towards AI, results, limitations, and conclusions. The data supporting the findings of this study are available from the corresponding author upon reasonable request.

### 2.4. Statistical Analysis

We identified factors associated with physician buy-in using inductive thematic analysis. Narrative data from the included studies (e.g., reported barriers, facilitators, and contextual explanations) were openly coded by three independent reviewers (GH, AS, and PH). Codes were iteratively categorized into broader groups and refined into themes through constant comparison until thematic saturation was reached, with discrepancies resolved through discussion. This process resulted in five overarching themes: (1) knowledge, (2) engagement, (3) legal, regulatory, and policy uncertainties for responsible use of AI, (4) workflow, and (5) patient-centeredness.

For studies that rated participant knowledge of AI into low and high levels, we assigned a knowledge score of 1–5 based on the criteria below (Table 1). Participants in studies with higher knowledge scores were assumed to have higher AI knowledge.

In addition to providing a qualitative description of the findings and categorizing studies based on knowledge score, we conducted a meta-analysis to estimate a pooled level of low, moderate, and high overall AI knowledge. Forest plots were employed to illustrate variability across studies, and subgroup differences were evaluated using Chi-square tests. The pooled rates of either knowledge level showed substantial heterogeneity, prompting the use of a random effects model. Both heterogeneity and subgroup analyses were conducted on prevalence rates transformed via the Logit transformation method to stabilize variance, although original prevalence values were retained in tables and figures for clarity. Heterogeneity was assessed using the I^2^ statistic and the DerSimonian and Laird estimator. An I^2^ value exceeding 75% was considered indicative of high heterogeneity. To assess publication bias, funnel plots were visually inspected, and Egger’s test was applied, with *p*-values below 0.05 indicating significant bias. All meta-analyses and visualizations, including forest and funnel plots, were carried out in R version 4.4.3 using the “metafor” and “meta” packages.

## 3. Results

### 3.1. Search Results

**Figure 1:**
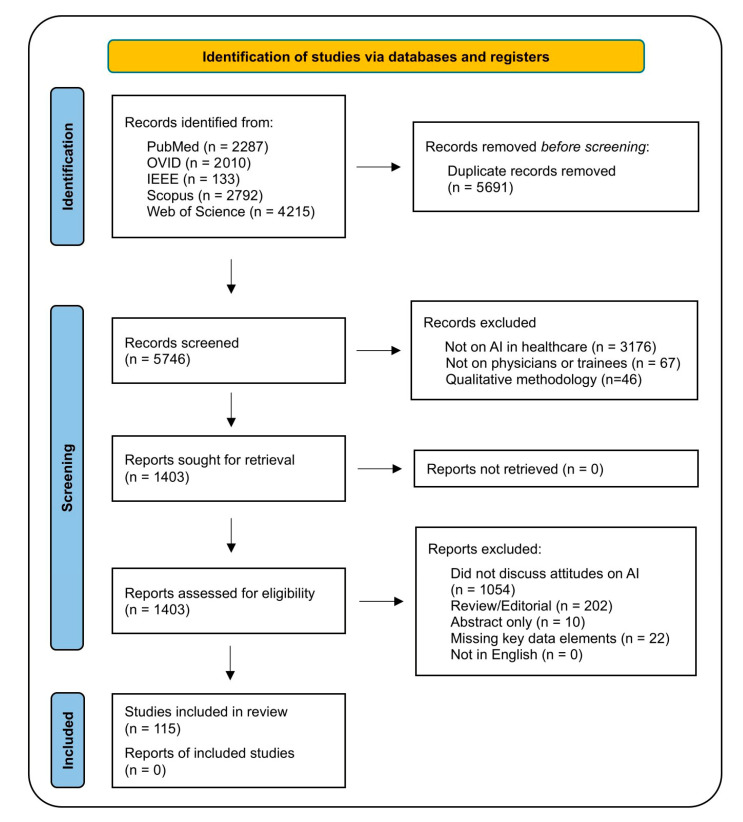
PRISMA Flow Diagram.

The search yielded 11,437 studies. After the removal of duplicates, 5746 results were screened for relevance based on title and abstracts. Results that did not discuss AI solutions in healthcare (*n* = 3176), did not evaluate physicians and/or medical students (*n* = 67), or used qualitative methodology (*n* = 46) were removed. Screening identified 1403 results for retrieval and eligibility. We excluded studies that did not discuss attitudes (*n* = 1054), reviews/editorials (*n* = 202), abstracts (*n* = 10), and/or had missing key data elements (*n* = 22). A flow diagram of this process is provided as Figure 1.

### 3.2. Study Characteristics

**Figure 2:**
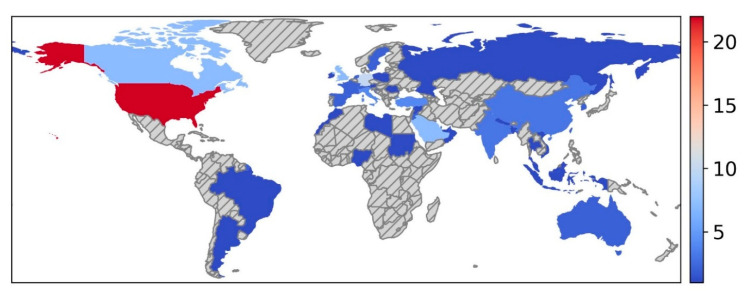
World map highlighting the 42 countries represented in the included studies, based on senior author affiliation. The scale indicates the number of papers contributed by each country.

**Figure 3:**
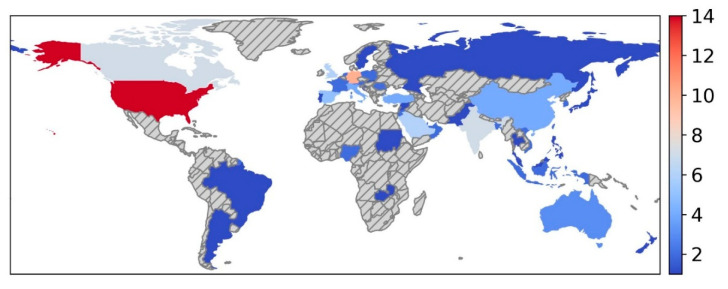
World map highlighting the 62 countries represented in the included studies, based on the nationality of survey participants. The scale reflects the number of papers from each country.

The publication dates ranged from 2018 to 2024. The senior authors represented 42 countries (Figure 2), and participants were recruited from more than 62 countries (Figure 3). All studies were cross-sectional. Among the included studies, 100 (87%) studies collected data from participants at multiple centers, and 15 (13%) studies collected data from participants at a single center. Regarding AI attitudes, 101 (88%) studies evaluated the physicians’ attitudes towards general AI applications in healthcare, whereas 14 (12%) evaluated attitudes towards a specific AI application, including diagnostic support for imaging modalities such as mammography, colonoscopy, endoscopy, CT lung, and chest X-Ray.

### 3.3. Design of Included Studies

All included studies (*n* = 115) administered questionnaires that were analyzed with quantitative methodologies to examine the attitudes of physicians and/or medical trainees towards AI in healthcare.

### 3.4. Reported Study Bias and Limitations

Assessment of risk of bias and limitations of the studies reviewed is presented in the Supplementary Material. Several studies reported (*n* = 99) the risk of participation bias, as people with extremely positive or extremely negative attitudes towards AI, more advanced knowledge of AI, or more utilization of AI might be more inclined to respond to survey recruitment. All studies utilized convenience sampling via a professional society or institutional listserv, which could introduce sampling bias and self-selection bias. One study also utilized snowball sampling, which could introduce non-randomization bias.

### 3.5. Reported Overarching Study Findings

Most (*n* = 60, 52%) studies concluded that physicians and trainees are “cautiously optimistic” about AI in healthcare (see Supplementary Material). Our review of the literature revealed five factors associated with physician and trainee buy-in, using inductive thematic analysis, which are presented below in order of prominence from the literature. We have categorized these factors below: (1) knowledge, (2) engagement, (3) legal, regulatory, and policy uncertainties for responsible use of AI, (4) workflow, and (5) patient-centeredness.

### 3.6. Knowledge

**Figure 4:**
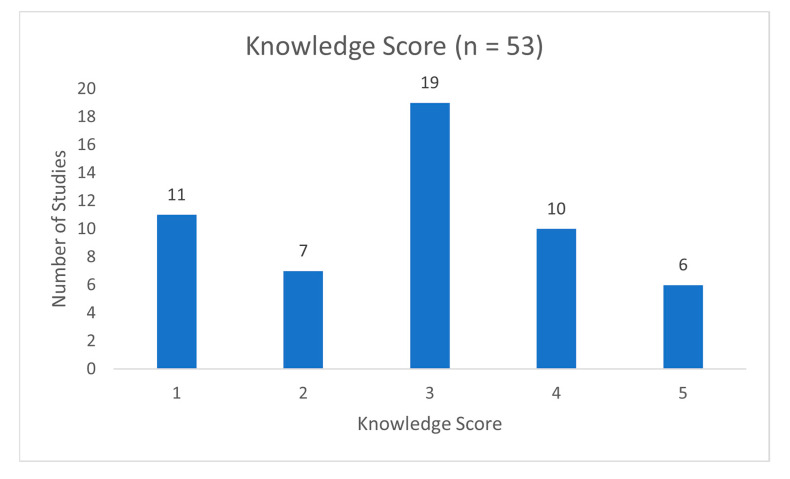
Assigned knowledge scores for *n* = 53 studies, where a score of 1 indicates that the majority of survey respondents had low AI knowledge, and a score of 5 indicates that the majority had high AI knowledge.

Objective or subjective approaches were utilized by 76 studies to assess the AI knowledge of the participants. Objective assessments (*n* = 10, 12.7%) rated participant knowledge by percent of correct responses to statements about how AI is defined and utilized (see Supplementary Material). Subjective assessments (*n* = 66, 87.3%) were measured by participants’ self-rated knowledge of AI (see Supplementary Material). To categorize studies (Figure 4), knowledge scores from 1 to 5 were assigned to studies rating respondents’ AI knowledge into low or high levels (*n* = 53), either using an objective or subjective assessment (see Supplementary Material). Of the studies, 19 (35.8%) indicated that about half of the respondents had low self-rated or investigator-rated AI knowledge (see Supplementary Material). Only 6 (11%) studies reported that at least 75% of respondents had high self-rated or investigator-rated AI knowledge [12,13,14,15,16,17].

Of 76 studies evaluating knowledge, 55 studies (72%) evaluated the depth of AI knowledge among physicians and trainees. These studies consistently concluded that most participant possess only moderate conceptual knowledge of AI, and their technical knowledge is usually limited (see Supplementary Material). Twenty-one studies (18%) evaluated the association between knowledge of AI and attitudes towards AI. A high level of conceptual knowledge of AI was reported to be associated with greater receptiveness to AI implementation in medicine (see Supplementary Material). A strong demand for additional AI education was reported in 42 studies (55.3%) by both trainees and physicians, spanning medical school curricula, residency lectures, Continuing Medical Education (CME) activities, and workplace training programs. (see Supplementary Material).

Based on our random-effect model meta-analysis of studies reporting detailed rates of low, medium, or high overall AI knowledge among their participants, the overall estimated rates were 36.3% (*n* = 40, I^2^ = 98.8%, Figures S1–S3) for low, 48% (*n* = 23, I^2^ = 97.5%, Figures S4–S6) for medium, and 19.6% (*n* = 28, I^2^ = 97.5%, Figures S7–S9) for high AI knowledge. In another estimate, the low-to-medium overall AI knowledge was 79.3% (*n* = 28, I^2^ = 97.7%, Figures S10–S12) and the medium-to-high overall AI knowledge was 62.8% (*n* = 40, I^2^ = 98.8%, Figures S13–S15). Based on I^2^ values, substantial heterogeneity was observed across all studies, prompting further assessment of AI knowledge levels through subgroup meta-analysis. However, subgroup analyses by participant type (physician vs. physician-trainees; Figures S1, S4, S7, S10 and S13), country (Figures S2, S5, S8, S11 and S14), and participant specialty (Figures S3, S6, S9, S12 and S15) did not meaningfully reduce the heterogeneity (Table S1 summarizes results of subgroup analyses). Neither visual inspection of the funnel plots nor Egger’s tests revealed significant publication bias among the included studies; however, the interpretation should be made cautiously given the substantial heterogeneity across studies, and I^2^ values were close to 100% (Figure S16).

### 3.7. Engagement

Among 115 studies evaluating engagement, 50 articles (42%) demonstrated that it is critical to engage physicians and trainees in the early stages of AI design. Physicians and trainees expressed interest in AI solutions for clinical documentation, risk stratification and triage, and diagnostic support (see Supplementary Material).

Additionally, 2 studies (2%) investigated whether AI would affect trainee interest in radiology or pathology [18,19], and 21 (18%) studies explored whether physicians in these specialties would feel an increased threat to job status compared to other specialties with fewer AI applications (see Supplementary Material). However, of the studies published thus far, no consistent perspective on the effect of AI on trainee interest in radiology and pathology has been found. Although specialties, such as radiology and pathology, are more likely to be affected by AI applications, 15 studies (13%) reported that physicians in these specialties were not significantly more concerned with job replacement compared to physicians in other medicine and surgical specialties, and have greater conceptual and technical knowledge, as well as awareness of AI. (see Supplementary Material).

One study evaluated the use of an AI-based clinical decision support tool (i.e., Glass AI) in medical education [19]. Although first-year medical students found Glass AI helpful for increasing their confidence in recognizing diagnostic patterns, they felt that the tool was inadequate in explaining physiology concepts. Furthermore, participants felt that human input was extremely important for successful AI implementation [19].

### 3.8. Legal, Regulatory, and Policy Uncertainties for Responsible Use of AI

Our review revealed a dearth of empirical data regarding legal issues, such as liability risks, appropriate apportionment of liability, regulatory uncertainty, or policy development. Only three (3%) studies [20,21,22]—from distinct geographic locations and, thus, different legal jurisdictions—mentioned “malpractice.” General sentiment reported from Germany [22,23] suggests physicians expect the use of AI to offer protection from liability and lower malpractice risks (e.g., by improving documentation in medical decision-making). By contrast, perspectives reported from Canada [24] reveal general concerns about ethical, legal, and social issues regarding the use of AI in primary and digital healthcare are “non-starters.” Those reported from the Netherlands [25] reveal that the “uncertainty about laws and regulations (responsibility)” was a major disadvantage of AI among gastrointestinal physicians.

Data regarding who should be legally responsible for mistakes or poor outcomes from using AI show no clear consensus [21,22,26,27]. Some data (such as a study in Oman) suggest shared liability among physician-users and manufacturers of AI tools. Other data from an international survey suggested primary responsibility should be borne by physicians-users [28]. Yet other data (such as an Australian study of referring doctors for AI-based radiology) suggest that vicarious liability (i.e., liability of the hospitals or healthcare systems rather than the physicians) might be appropriate [21]. Perspectives of physician-users as to whether manufacturers should share in liability risks have been reported to correlate with increased age or years of experience of the physician-users studied [28].

No common interpretation emerged from the studies reviewed regarding the appropriate implementation of AI in the midst of legal uncertainty. While some scholars suggested legal and regulatory uncertainties must be “sufficiently addressed” [21] or “authoritatively resolved” [20] *before* AI is implemented in clinical settings, others [29] suggested that, in the “absence of regulatory standards”, AI should only be piloted in well-resourced facilities that might be better equipped to manage “challenges related to change, such as formulating new regulatory standards, defining responsibilities, and determining accountability.” [29].

### 3.9. Workflow

Thirteen (*n* = 13, 11%) studies cited workflow impediments as a barrier to physician buy-in (see Supplementary Material). In one study, only 36% of surveyed physicians considered AI easy to implement [30]. Eastwood et al. reported that emergency department physicians wanted AI solutions to be integrated within existing digital workflows [31]. However, in two separate studies, many participants felt that it would be difficult to integrate AI within existing IT infrastructure [32,33]. Other workflow impediments that were mentioned by studies (*n* = 13, 11%) included excessive clicks and alert pop-ups, lack of integration with EHR, lack of technical support for malfunctions, and inability to advance screens if alerts are not addressed (see Supplementary Material).

Another challenge in the AI workflow is the disagreement between physicians and AI recommendations. In situations where a physician disagrees with AI-based recommendations, eighteen (*n* = 18, 16%) studies showed a majority (64–86%) of physician and trainee respondents agreed that physicians would defer to their judgment or consult another senior physician (see Supplementary Material). Eighty-four percent (84%) of radiologists surveyed by Ce et al. asserted that final review of AI-generated imaging reads by radiologists was fundamental to the workflow [34].

### 3.10. Patient-Centeredness

Twenty-one (*n* = 21) studies asked participants about how AI will affect the physician-patient relationship (see Supplementary Material). Findings revealed that respondents had split opinions, with 53–57% of respondents across these studies believing that AI could negatively impact the patient-physician relationship by reducing face-to-face cues and decreasing trust in physicians. In addition, physicians and trainees overwhelmingly agreed that AI could not provide empathetic care. In contrast, some respondents felt that AI could benefit patient communication [31,35,36]. From another point of view, psychiatrists surveyed by Blease et al. highlighted that AI could strengthen dialogue by helping patients be better prepared at appointments [37].

In another study, 52% respondents were concerned that tools like AI waiting rooms that are designed to assist with the check-in and triage process could diminish the personal touches of healthcare and negatively impact patient satisfaction [17]. On the other hand, another study reported that dermatologists view AI as most useful as administrative assistants rather than as mediators between healthcare professionals and patients [38].

## 4. Discussion

Our systematic review of 115 studies showed that physicians and trainees are cautiously optimistic about AI’s applications in healthcare; however, several factors continue to limit buy-in. Insufficient conceptual knowledge of AI and limited engagement in early stages of AI design were frequently reported barriers. Additional concerns related to legal, regulatory, and policy uncertainties surrounding responsible AI use, potential workflow disruptions, and risks to patient-centeredness further contributed to hesitancy among physicians and trainees. Based on our analysis, we present and discuss a set of five actionable recommendations to facilitate physician and trainee buy-in to AI implementation in a range of clinical specialties.

### 4.1. Establish Standards for Education and Workforce Training of AI-Based Tools

Our quantitative meta-analysis estimated that only 19.6% of physicians and physician-trainees demonstrated high overall AI knowledge, whereas up to 36.3% exhibited low overall knowledge. Several included studies further characterized the depth of AI knowledge by distinguishing conceptual and technical understanding. Although the available data were insufficient to support a quantitative synthesis, the findings from these studies consistently suggest that physicians and trainees who report having AI knowledge predominantly possess conceptual rather than technical understanding. Conceptual knowledge of AI refers to a broad, nonspecific understanding of AI as a tool capable of processing data and generating recommendations. On the other hand, technical knowledge of AI reflects a deeper understanding of how AI models process data, how AI technology is updated, and how ML-based CDSS differ from conventional CDSS.

Moreover, studies evaluating the depth of AI knowledge among physicians and physician-trainees reported that greater technical familiarity was associated with higher trust and receptiveness toward AI (see Supplementary Material). However, this association was not examined in our meta-analysis and therefore cannot be confirmed quantitatively.

Although reviewed studies also indicate that physicians and trainees highly value education and workforce training delivered through conferences, resident didactics, and curriculum electives, the specific type of education/training required to meaningfully influence their intention and willingness to use AI remains unclear. It is uncertain whether advanced technical understanding of the AI’s inner workings is feasible or necessary to enhance physician buy-in. However, one study that categorized physicians into behavioral profiles—including “AI-educated,” “AI-positive, and “AI-skeptical”—reported that intention to use AI was influenced both by greater technical knowledge and by a stronger conceptual understanding of the value that AI adds to clinical practice [39]. Conversely, participants evaluating an AI-based sepsis alert expressed a preference for ML-powered CDSS over traditional CDSS and reported curiosity about the inner workings of ML, yet they indicated that understanding the system’s logic in an individual case would not change their clinical decision-making [40].

Given the limited time available for physician-trainees for continuing medical education (CME) activities, it is essential to focus on educational approaches that efficiently enhance both the intention and ability of physicians to use AI. Effective education can help bridge the gap between envisioned and actual AI applications, and equip physicians to adopt a discerning stance on issues such as legal uncertainty and algorithmic transparency [20,41,42]. Accordingly, Terry et al. (2022) [24] concluded that “effective deployment of AI tools requires emphasizing the value AI brings rather than its mechanics”.

### 4.2. Engage Physicians and Trainees in the Early Stages of AI Design and Development

Collaborating with physicians and trainees during AI development helps ensure that AI tools are effectively tailored to end-users and address barriers to adoption. Both physicians and trainees view AI positively, particularly as a support tool for administrative tasks and triage of large volumes of patient data, rather than as a replacement for clinical judgment in patient care. Including physician input is therefore essential to ensure that AI tools align with current standards of clinical decision-making and integrate smoothly into existing workflow [41].

In addition to the benefits of involving physicians and trainees in AI development, some studies have highlighted that AI could support clinicians with less experience or resources during medical education or practice. Some trainees identified that AI tools like ChatGPT could be used to generate practice questions, simulated patient scenarios, and illness scripts; however, only one of the included studies directly evaluated the application of AI specifically for medical education [43]. Organizations such as the Association of American Medical Colleges (AAMC) and the International Advisory Committee of Artificial Intelligence (IACAI) have begun publishing guidelines for integrating AI in medical education [44,45]. Further research is required to understand how medical trainees utilize AI and their preferences for learning about AI, in order to inform the development of evidence-based guidelines for its use.

### 4.3. Collaborate with Physicians and Trainees to Clarify Legal Uncertainties

As primary end-users, physicians need to be involved in the development of standards for role clarity, liability, and malpractice, and algorithmic transparency. Very few studies explored malpractice (*n* = 3) or liability (*n* = 4). The limited coverage comes from data scattered across distinct geographic locations that might not offer generalizable or transferable insights, with study samples drawn from clinicians in Australia [21], Canada [24], China [46], Germany [23], the Netherlands [25], Oman [25], and internationally [29]. The establishment of best practices for physician versus AI role definition, liability, and malpractice, and algorithmic transparency will reduce perceived threats to autonomy and expand the scenarios in which physicians feel comfortable using AI tools to support their clinical decision-making [47,48]. Our review shows that physicians tend to be divided on whether physicians or AI developers should be liable for AI-aided decisions. However, in times when their decision conflicts with the AI-generated recommendation, the majority of physicians would rely on their own or their colleague’s judgment. Further research needs to distinguish between moral/professional responsibility for patient outcomes and malpractice standards [47,49].

### 4.4. Draw upon Literature on the Implementation of Clinical Decision Support Systems to Minimize Technical Workflow Barriers

Workflow impediments were an often-cited barrier to physician buy-in. These barriers parallel physician frustrations with traditional CDSS and EHR technology implementations, such as excessive clicks and alert pop-ups, lack of integration with EHR, lack of technical support for malfunctions, and inability to advance screens if alerts are not addressed [50,51]. Since there is a large body of implementation science literature for CDSS and EHR technology, using lessons learned from these technologies can improve the technical implementation of AI tools. For example, the adoption of new CDSS and EHR technologies is improved when there are limited repetitive active CDSS alerts that require clinicians to interact with the alert to continue navigating the EHR [52]. Limiting workflow disruptions helps preserve physician autonomy and improve acceptance of new technology. Furthermore, prioritizing alerts for high specificity versus high sensitivity disease contexts is important to maximize the relevance of the alert. Finally, this literature emphasizes the importance of institutional infrastructure in supporting technical challenges with implementation.

### 4.5. Incorporate Principles of Patient-Centered Communication into AI-Based Tools

Our final actionable recommendation is in response to physician concerns that AI might compromise the physician-patient relationship by disrupting direct communication. Quality improvement and financial incentives increasingly reward physicians for delivering patient-centered services. Therefore, AI tools should align with the patient-centered frameworks of care and augment the ability of physicians to achieve these goals. A growing body of literature is examining patients’ attitudes toward AI-based healthcare, which can serve as an important guide for implementing patient-centered AI tools [1,2,3,4]. This step is essential because if physicians perceive that AI could undermine their progress towards improved patient-centered outcomes and related incentives, securing their buy-in will be challenging.

### 4.6. Limitations

This review has some limitations. Each study utilized different questionnaires to assess knowledge and attitudes, and therefore, there were inconsistent definitions of low, medium, and high knowledge, which may affect comparability. Due to the range of methodologies used in individual studies, our meta-analysis can only suggest trends in overall knowledge of AI rather than precise measurements. Several studies use terms such as “cautiously optimistic” to summarize attitudes towards AI. However, the individual measures and results of these studies differ significantly. The development of a validated study tool might facilitate more precise comparisons across subgroups of participants; however, even in such instances, careful attention would need to be given to confounding effects of jurisdictional factors and other cultural conditions shaping those perspectives.

## 5. Conclusions

Our review of the literature indicates that physicians and trainees are receptive to AI in medicine. However, there are numerous barriers to buy-in, which include low operating knowledge, concerns about job replacement and workforce de-skilling, and the lack of established regulatory standards. We have offered a set of five actionable recommendations regarding the inclusion of physician stakeholders in AI development and the establishment of standards for education, liability, workflow, and patient-centeredness.

## Figures and Tables

**Table 1:** Criteria used to score studies based on participants’ AI knowledge.

Score	Criteria
1	76–100% of participants had low self-rated or investigator-rated knowledge of AI
2	56–75% of participants had low self-rated or investigator-rated knowledge of AI
3	45–55% of participants had low self-rated or investigator-rated knowledge of AI
4	56–75% of participants had high self-rated or investigator-rated knowledge of AI
5	76–100% of participants had high self-rated or investigator-rated knowledge of AI

## Data Availability

The original contributions presented in this study are included in the article/Supplementary Material. Further inquiries can be directed to the corresponding author.

## Supplementary Materials

The following supporting information can be downloaded at: https://www.mdpi.com/article/10.3390/bioengineering12121363/s1. Table S1. The Summary of Sub-group analyses for Estimating Pooled Rates of Low, Medium, and High AI Knowledge Across the Included Studies, Stratified by the Type of the Participants, Country, and Participants’ Specialty. Figure S1. Rates Of Low AI Knowledge Across the Included Studies, Stratified by the Type of the Participants. Each study’s effect size represents the percentage of participants with low AI knowledge in that study. The dotted line indicates the overall estimated rate of low AI knowledge. Figure S2. Rates Of Low AI Knowledge Across the Included Studies, Stratified by the Country of the Participants. Each study’s effect size represents the percentage of participants with low AI knowledge in that study. The dotted line indicates the overall estimated rate of low AI knowledge. Figure S3. Rates Of Low AI Knowledge Across the Included Studies, Stratified by the Specialty of the Participants. Each study’s effect size represents the percentage of participants with low AI knowledge in that study. The dotted line indicates the overall estimated rate of low AI knowledge. Figure S4. Rates Of Medium AI Knowledge Across the Included Studies, Stratified by the Type of the Participants. Each study’s effect size represents the percentage of participants with low AI knowledge in that study. The dotted line indicates the overall estimated rate of medium AI knowledge. Figure S5. Rates Of Medium AI Knowledge Across the Included Studies, Stratified by the Country of the Participants. Each study’s effect size represents the percentage of participants with medium AI knowledge in that study. The dotted line indicates the overall estimated rate of medium AI knowledge. Figure S6. Rates Of Medium AI Knowledge Across the Included Studies, Stratified by the Specialty of the Participants. Each study’s effect size represents the percentage of participants with medium AI knowledge in that study. The dotted line indicates the overall estimated rate of medium AI knowledge. Figure S7. Rates Of High AI Knowledge Across the Included Studies, Stratified by the Type of the Participants. Each study’s effect size represents the percentage of participants with high AI knowledge in that study. The dotted line indicates the overall estimated rate of high AI knowledge. Figure S8. Rates Of High AI Knowledge Across the Included Studies, Stratified by the Country of the Participants. Each study’s effect size represents the percentage of participants with high AI knowledge in that study. The dotted line indicates the overall estimated rate of high AI knowledge. Figure S9. Rates Of High AI Knowledge Across the Included Studies, Stratified by the Specialty of the Participants. Each study’s effect size represents the percentage of participants with high AI knowledge in that study. The dotted line indicates the overall estimated rate of high AI knowledge. Figure S10. Rates Of Low or Medium AI Knowledge Across the Included Studies, Stratified by the Type of the Participants. Each study’s effect size represents the percentage of participants with low or medium AI knowledge in that study. The dotted line indicates the overall estimated rate of low or medium AI knowledge. Figure S11. Rates Of Low or Medium AI Knowledge Across the Included Studies, Stratified by the Country of the Participants. Each study’s effect size represents the percentage of participants with low or medium AI knowledge in that study. The dotted line indicates the overall estimated rate of low or medium AI knowledge. Figure S12. Rates Of Low or Medium AI Knowledge Across the Included Studies, Stratified by the Specialty of the Participants. Each study’s effect size represents the percentage of participants with low or medium AI knowledge in that study. The dotted line indicates the overall estimated rate of low or medium AI knowledge. Figure S13. Rates Of Medium or High AI Knowledge Across the Included Studies, Stratified by the Type of the Participants. Each study’s effect size represents the percentage of participants with medium or high AI knowledge in that study. The dotted line indicates the overall estimated rate of medium or high AI knowledge. Figure S14. Rates Of Medium or High AI Knowledge Across the Included Studies, Stratified by the Country of the Participants. Each study’s effect size represents the percentage of participants with medium or high AI knowledge in that study. The dotted line indicates the overall estimated rate of medium or high AI knowledge. Figure S15. Rates Of Medium or High AI Knowledge Across the Included Studies, Stratified by the Specialty of the Participants. Each study’s effect size represents the percentage of participants with medium or high AI knowledge in that study. The dotted line indicates the overall estimated rate of medium or high AI knowledge. Figure S16. Funnel Plots Evaluating the Risk of Publication Bias Among the Included Studies. Results of Egger’s tests are indicated below each funnel plot.

## Abbreviations

The following abbreviations are used in this manuscript:

AI	Artificial intelligence
AAMC	Association of American Medical Colleges
CDSS	Clinical decision support systems
CME	Continuing Medical Education
DL	Deep learning
IACAI	International Advisory Committee of Artificial Intelligence
ML	Machine learning
NIH	National Institute of Health
NINDS	National Institute of Neurological Disorders and Stroke
PRISMA	Preferred Reporting Items for Systematic Reviews and Meta-Analyses 2020 Checklist
UK	United Kingdom
USA	United States of America
