# Supplementary material for: Translating AI to the Bedside with Physician Buy-In: Recommendations from a Meta-Analysis and Systematic Review of the Literature
Source: Bioengineering (Basel). 2025 Dec 16;12(12):1363. doi: 10.3390/bioengineering12121363 (PMC12729787; doi:10.3390/bioengineering12121363)
Supplement: Supplementary file 1 [file bioengineering-12-01363-s001.zip › bioengineering-3977089-supplementary.pdf]

Table S1. The Summary of Sub-group analyses for Estimating Pooled Rates of Low, Medium, and High AI Knowledge Across the Included Studies, Stratified by the Type of the Participants, Country, and Participants' Specialty.

	Subgroup	Low knowledge	Medium Knowledge	High Knowledge	Low-to-Medium knowledge	Medium-to-High Knowledge
Participant Type	Physician	39.3% [31.5-47.7]	38% [30.5-46.1]	23.3% [13.9-36.3]	76% [62.9-85.5]	60% [51.7-67.8]
	Student	15.2% [3-50.8]	74.9% [59.2-86]	12.8% [6.9-36.3]	87.2% [77.1-93.3]	84.9% [49.2-97]
	Physician + Student	22.8% [18.2-28.2]	63.7% [58.4-68.7]	8.7% [2.1-29.5]	91.3% [70.5-97.9]	77.2% [71.81.8]
	Unknown	39.9% [27.4-53.8]	48.5% [41.5-55.6]	21% [16.3-26.5]	77.2% [70.7-82.6]	58.7% [44.9-71.3]
Country	Asia	27.7% [18.7-34.1]	57.4% [44.5-69.3]	17.8% [11-27.3]	81.6% [71.9-88.4]	73.7% [65.2-80.6]
	Europe	40.5% [26.7-64.7]	37.7% [24.8-52.7]	16.1% [8.4-28.7]	81.7% [67-90.8]	52.9% [34-71.1]
	North America	46.9% [32.9-61.4]	50.7% [42.3-59]	13.2% [3.8-37.1]	86.6% [61.8-96.3]	53% [38.6-67]
	Pacific	27.7% [24.2-31.4]	47.6% [43.7-51.6]	44% [12.1-81.7]	56% [18.3-87.9]	72.3% [68.6-75.8]
	International	28.2% [19.4-38.9]	49.5% [40.5-58.4]	30.1% [22-3-39.1]	70% [60.9-77.8]	71.9% [61-80.7]
	Unknown	54.8% [48.1-61.3]	-	-	-	45.2% [38.7-51.9]
Specialty	Family/internal Medicine	44.9% [24.3-67.4]	34.9% [25.1-46.1]	13.5% [5-31.8]	85.3% [67.5-94.2]	53.3% [31.8-73.7]
	Anesthesiology	29.8% [16.2-48.2]	54.3% [51.3-57.3]	50% [11.2-88.8]	50% [11.2-88.8]	70.2% [51.8-83.8]
	Emergency Medicine	30.4% [23.6-37.9]	45.6% [38-53.4]	23.4% [17.3-30.5]	76% [68.9-82.2]	69% [61.5-75.8]
	Radiology	36.5% [18.9-58.7]	25.3% [21.8-29.1]	47.9% [43.7-52.1]	52.1% [47.9-56.3]	63.5% [41.3-81.1]
	Orthodontics	30% [21.2-40]	66% [56.8-75.2]	4% [1.1-9.9]	96% [90.1-98.9]	70% [60-78.8]
	Mixed	40.6% [26.9-55.9]	41% [32.5-50.1]	17% [1.2-77]	83% [23-98.8]	59.4% [44.1-73.1]
	Unknown	34.3% [24.4-45.8]	54.7% [45.8-63.3]	19.1% [14.8-24.2]	79.6% [73.4-84.7]	64.7% [53-74.8]

Study	Participants	Specialty	Country	Case N	Total N	Effect Size	95%CI	w.random
**Participant = Unknown**								
Al-Ali 2023	Unknown	Unknown	Saudi Arabia	31	72	0.431	[0.314; 0.553]	2.4%
Al-Medfa 2023	Unknown	Unknown	Bahrain	30	122	0.246	[0.172; 0.332]	2.4%
Al-Zaabi 2023	Unknown	Unknown	Oman	91	293	0.311	[0.258; 0.367]	2.5%
Bisdas 2021	Unknown	Unknown	International	938	3033	0.309	[0.293; 0.326]	2.5%
Catalina 2023	Unknown	Unknown	Spain	43	301	0.143	[0.105; 0.188]	2.5%
Choi 2023	Unknown	Unknown	Korea	20	73	0.274	[0.176; 0.391]	2.4%
Civaner 2022	Unknown	Unknown	Turkey	2827	3008	0.940	[0.931; 0.948]	2.5%
Eschert 2022	Unknown	Unknown	Germany	137	302	0.454	[0.397; 0.512]	2.5%
Gunasekeran 2022	Unknown	Unknown	International	245	1107	0.221	[0.197; 0.247]	2.5%
Hogberg 2023	Unknown	Unknown	Sweden	17	47	0.362	[0.227; 0.515]	2.3%
Huisman 2021	Unknown	Unknown	Europe	305	1039	0.294	[0.266; 0.322]	2.5%
Jussupow 2022	Unknown	Unknown	Unknown	126	230	0.548	[0.481; 0.613]	2.5%
Liu 2022	Unknown	Unknown	USA	334	388	0.861	[0.822; 0.894]	2.5%
Pecqueux 2022	Unknown	Unknown	Germany	60	144	0.417	[0.335; 0.502]	2.5%
Pinto 2019	Unknown	Unknown	Germany	126	263	0.479	[0.417; 0.541]	2.5%
Pucchio 2022	Unknown	Unknown	Canada	304	486	0.626	[0.581; 0.669]	2.5%
Scheetz 2021	Unknown	Unknown	Australia	175	632	0.277	[0.242; 0.314]	2.5%
Shiang 2022	Unknown	Unknown	Isreal	4	15	0.267	[0.078; 0.551]	1.9%
Valikodath 2021	Unknown	Unknown	USA	7	80	0.087	[0.036; 0.172]	2.2%
**Random effects model**					**11635**	**0.399**	**[0.274; 0.538]**	**46.1%**
**Prediction interval**							**[0.044; 0.906]**	
Heterogeneity: I^2 = 99.3%, τ^2 = 1.5341, χ^2_{18} = 2493.97 (p < 0.001)								
**Participant = Physicians**								
Al Medfa 2023	Physicians	Mixed	Bahrain	30	166	0.181	[0.125; 0.248]	2.4%
Alexandrou 2024	Physicians	Family/Internal medicine	North America + Europe	79	521	0.152	[0.122; 0.185]	2.5%
Cai 2024	Physicians	Anesthesiology	China	87	396	0.220	[0.180; 0.264]	2.5%
Eastwood 2023	Physicians	Emergency medicine	Canada	52	171	0.304	[0.236; 0.379]	2.5%
Eltorai 2024	Physicians	Radiology	USA	101	211	0.479	[0.410; 0.548]	2.5%
Estrada Alamo 2024	Physicians	Anesthesiology	USA	418	1083	0.386	[0.357; 0.416]	2.5%
Hantel 2024	Physicians	Family/Internal medicine	USA	92	203	0.453	[0.383; 0.524]	2.5%
Iyengar 2023	Physicians	Mixed	Europe + Asia	66	125	0.528	[0.437; 0.618]	2.5%
McGenity 2024	Physicians	Family/Internal medicine	UK	29	42	0.690	[0.529; 0.824]	2.3%
Nishan 2024	Physicians	Mixed	Bangladesh	115	398	0.289	[0.245; 0.336]	2.5%
Orlova 2023	Physicians	Mixed	Russia	151	301	0.502	[0.444; 0.560]	2.5%
Radhwi 2024	Physicians	Mixed	Saudi Arabia	76	128	0.594	[0.503; 0.680]	2.5%
Sahin 2023	Physicians	Family/Internal medicine	Turkey	101	156	0.647	[0.567; 0.722]	2.5%
Savoia 2023	Physicians	Family/Internal medicine	Europe	158	239	0.661	[0.597; 0.721]	2.5%
Waheed 2024	Physicians	Family/Internal medicine	Qatar	22	108	0.204	[0.132; 0.292]	2.4%
Zanardo 2024	Physicians	Radiology	Europe	153	572	0.267	[0.232; 0.306]	2.5%
**Random effects model**					**4820**	**0.393**	**[0.315; 0.477]**	**39.4%**
**Prediction interval**							**[0.127; 0.743]**	
Heterogeneity: I^2 = 96.5%, τ^2 = 0.4605, χ^2_{15} = 424.31 (p < 0.001)								
**Participant = Phy+Stu**								
Dumitrascu 2024	Phy+Stu	Unknown	Romania	62	309	0.201	[0.157; 0.250]	2.5%
Mengi 2024	Phy+Stu	Orthodontics	India	30	100	0.300	[0.212; 0.400]	2.4%
Tangadulrat 2023	Phy+Stu	Unknown	Thailand	51	239	0.213	[0.163; 0.271]	2.5%
**Random effects model**					**648**	**0.228**	**[0.182; 0.282]**	**7.4%**
**Prediction interval**							**[0.097; 0.449]**	
Heterogeneity: I^2 = 54.5%, τ^2 = 0.0342, χ^2_2 = 4.39 (p = 0.11)								
**Participant = Students**								
Lugito 2024	Students	Unknown	Indonesia	9	650	0.014	[0.006; 0.026]	2.3%
Malesivc 2024	Students	Unknown	East Europe	395	1701	0.232	[0.212; 0.253]	2.5%
Robleto 2024	Students	Unknown	USA	40	72	0.556	[0.434; 0.673]	2.4%
**Random effects model**					**2423**	**0.152**	**[0.030; 0.508]**	**7.2%**
**Prediction interval**							**[0.000; 0.997]**	
Heterogeneity: I^2 = 98.3%, τ^2 = 2.3507, χ^2_2 = 119.43 (p < 0.001)								
**Random effects model**					**19526**	**0.363**	**[0.293; 0.439]**	**100.0%**
**Prediction interval**							**[0.065; 0.822]**	

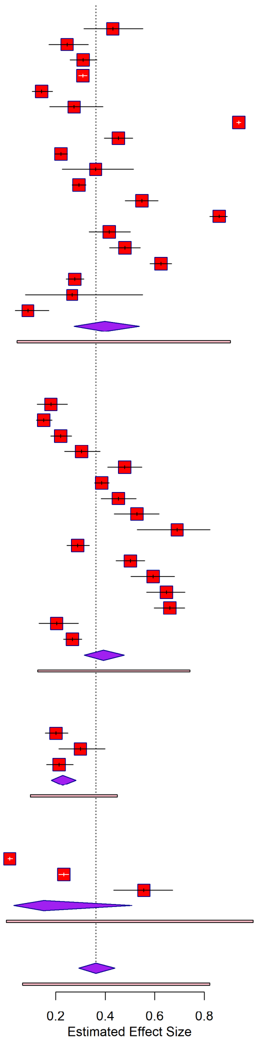

Heterogeneity: I^2 = 98.8%, τ^2 = 1.0473, χ^2_{40} = 3312.42 (p < 0.001)
Test for subgroup differences: χ^2_3 = 15.38, df = 3 (p = 0.002)

Figure S1. Rates Of **Low AI Knowledge** Across the Included Studies, Stratified by the **Type** of the Participants. Each study's effect size represents the percentage of participants with low AI knowledge in that study. The dotted line indicates the overall estimated rate of low AI knowledge.

Study	Participants	Specialty	Country	Case N	Total N	Effect Size	95%CI	w.random
**Country = Asia**								
Al-Ali 2023	Unknown	Unknown	Saudi Arabia	31	72	0.431	[0.314; 0.553]	2.4%
Al-Medfa 2023	Unknown	Unknown	Bahrain	30	122	0.246	[0.172; 0.332]	2.4%
Al-Zaabi 2023	Unknown	Unknown	Oman	91	293	0.311	[0.258; 0.367]	2.5%
Al Medfa 2023	Physicians	Mixed	Bahrain	30	166	0.181	[0.125; 0.248]	2.4%
Cai 2024	Physicians	Anesthesiology	China	87	396	0.220	[0.180; 0.264]	2.5%
Choi 2023	Unknown	Unknown	Korea	20	73	0.274	[0.176; 0.391]	2.4%
Lugito 2024	Students	Unknown	Indonesia	9	650	0.014	[0.006; 0.026]	2.3%
Mengi 2024	Phy+Stu	Orthodontics	India	30	100	0.300	[0.212; 0.400]	2.4%
Nishan 2024	Physicians	Mixed	Bangladesh	115	398	0.289	[0.245; 0.336]	2.5%
Orlova 2023	Physicians	Mixed	Russia	151	301	0.502	[0.444; 0.560]	2.5%
Radhwi 2024	Physicians	Mixed	Saudi Arabia	76	128	0.594	[0.503; 0.680]	2.5%
Shiang 2022	Unknown	Unknown	Isreal	4	15	0.267	[0.078; 0.551]	1.9%
Tangadulrat 2023	Phy+Stu	Unknown	Thailand	51	239	0.213	[0.163; 0.271]	2.5%
Waheed 2024	Physicians	Family/Internal medicine	Qatar	22	108	0.204	[0.132; 0.292]	2.4%
**Random effects model**					**3061**	**0.257**	**[0.187; 0.341]**	**33.5%**
**Prediction interval**							**[0.062; 0.643]**	
Heterogeneity: $I^2 = 94.8\%$, $\tau^2 = 0.5423$, $\chi^2_{13} = 249.02$ ($p < 0.001$)								
**Country = International**								
Alexandrou 2024	Physicians	Family/Internal medicine	North America + Europe	79	521	0.152	[0.122; 0.185]	2.5%
Bisdas 2021	Unknown	Unknown	International	938	3033	0.309	[0.293; 0.326]	2.5%
Gunasekeran 2022	Unknown	Unknown	International	245	1107	0.221	[0.197; 0.247]	2.5%
Iyengar 2023	Physicians	Mixed	Europe + Asia	66	125	0.528	[0.437; 0.618]	2.5%
**Random effects model**					**4786**	**0.282**	**[0.194; 0.389]**	**10.0%**
**Prediction interval**							**[0.065; 0.688]**	
Heterogeneity: $I^2 = 97.2\%$, $\tau^2 = 0.2336$, $\chi^2_{3} = 106.74$ ($p < 0.001$)								
**Country = Europe**								
Catalina 2023	Unknown	Unknown	Spain	43	301	0.143	[0.105; 0.188]	2.5%
Civaner 2022	Unknown	Unknown	Turkey	2827	3008	0.940	[0.931; 0.948]	2.5%
Dumitrascu 2024	Phy+Stu	Unknown	Romania	62	309	0.201	[0.157; 0.250]	2.5%
Eschert 2022	Unknown	Unknown	Germany	137	302	0.454	[0.397; 0.512]	2.5%
Hogberg 2023	Unknown	Unknown	Sweden	17	47	0.362	[0.227; 0.515]	2.3%
Huisman 2021	Unknown	Unknown	Europe	305	1039	0.294	[0.266; 0.322]	2.5%
Malesivc 2024	Students	Unknown	East Europe	395	1701	0.232	[0.212; 0.253]	2.5%
McGenity 2024	Physicians	Family/Internal medicine	UK	29	42	0.690	[0.529; 0.824]	2.3%
Pecqueux 2022	Unknown	Unknown	Germany	60	144	0.417	[0.335; 0.502]	2.5%
Pinto 2019	Unknown	Unknown	Germany	126	263	0.479	[0.417; 0.541]	2.5%
Sahin 2023	Physicians	Family/Internal medicine	Turkey	101	156	0.647	[0.567; 0.722]	2.5%
Savoia 2023	Physicians	Family/Internal medicine	Europe	158	239	0.661	[0.597; 0.721]	2.5%
Zanardo 2024	Physicians	Radiology	Europe	153	572	0.267	[0.232; 0.306]	2.5%
**Random effects model**					**8123**	**0.450**	**[0.267; 0.647]**	**32.0%**
**Prediction interval**							**[0.028; 0.958]**	
Heterogeneity: $I^2 = 99.5\%$, $\tau^2 = 2.1699$, $\chi^2_{12} = 2185.86$ ($p < 0.001$)								
**Country = N America**								
Eastwood 2023	Physicians	Emergency medicine	Canada	52	171	0.304	[0.236; 0.379]	2.5%
Eltorai 2024	Physicians	Radiology	USA	101	211	0.479	[0.410; 0.548]	2.5%
Estrada Alamo 2024	Physicians	Anesthesiology	USA	418	1083	0.386	[0.357; 0.416]	2.5%
Hantel 2024	Physicians	Family/Internal medicine	USA	92	203	0.453	[0.383; 0.524]	2.5%
Liu 2022	Unknown	Unknown	USA	334	388	0.861	[0.822; 0.894]	2.5%
Pucchio 2022	Unknown	Unknown	Canada	304	486	0.626	[0.581; 0.669]	2.5%
Robleto 2024	Students	Unknown	USA	40	72	0.556	[0.434; 0.673]	2.4%
Valikodath 2021	Unknown	Unknown	USA	7	80	0.087	[0.036; 0.172]	2.2%
**Random effects model**					**2694**	**0.469**	**[0.329; 0.614]**	**19.5%**
**Prediction interval**							**[0.099; 0.876]**	
Heterogeneity: $I^2 = 97.7\%$, $\tau^2 = 0.6856$, $\chi^2_{7} = 300.94$ ($p < 0.001$)								
**Country = Unknown**								
Jussupow 2022	Unknown	Unknown	Unknown	126	230	0.548	[0.481; 0.613]	2.5%
**Country = Pacific**								
Scheetz 2021	Unknown	Unknown	Australia	175	632	0.277	[0.242; 0.314]	2.5%
**Random effects model**					**19526**	**0.363**	**[0.293; 0.439]**	**100.0%**
**Prediction interval**							**[0.065; 0.822]**	

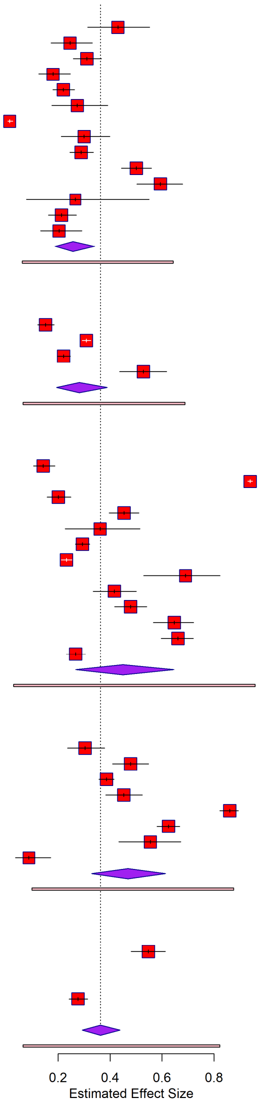

Heterogeneity: $I^2 = 98.8\%$, $\tau^2 = 1.0473$, $\chi^2_{40} = 3312.42$ ($p < 0.001$)
Test for subgroup differences: $\chi^2_{5} = 62.04$, df = 5 ($p < 0.001$)

Figure S2. Rates Of **Low AI Knowledge** Across the Included Studies, Stratified by the **Country** of the Participants. Each study's effect size represents the percentage of participants with low AI knowledge in that study. The dotted line indicates the overall estimated rate of low AI knowledge.

Study	Participants	Specialty	Country	Case N	Total N	Effect Size	95%CI	w.random
**Specialty = Unknown**								
Al-Ali 2023	Unknown	Unknown	Saudi Arabia	31	72	0.431	[0.314; 0.553]	2.4%
Al-Medfa 2023	Unknown	Unknown	Bahrain	30	122	0.246	[0.172; 0.332]	2.4%
Al-Zaabi 2023	Unknown	Unknown	Oman	91	293	0.311	[0.258; 0.367]	2.5%
Bisdas 2021	Unknown	Unknown	International	938	3033	0.309	[0.293; 0.326]	2.5%
Catalina 2023	Unknown	Unknown	Spain	43	301	0.143	[0.105; 0.188]	2.5%
Choi 2023	Unknown	Unknown	Korea	20	73	0.274	[0.176; 0.391]	2.4%
Civaner 2022	Unknown	Unknown	Turkey	2827	3008	0.940	[0.931; 0.948]	2.5%
Dumitrascu 2024	Phy+Stu	Unknown	Romania	62	309	0.201	[0.157; 0.250]	2.5%
Eschert 2022	Unknown	Unknown	Germany	137	302	0.454	[0.397; 0.512]	2.5%
Gunasekeran 2022	Unknown	Unknown	International	245	1107	0.221	[0.197; 0.247]	2.5%
Hogberg 2023	Unknown	Unknown	Sweden	17	47	0.362	[0.227; 0.515]	2.3%
Huisman 2021	Unknown	Unknown	Europe	305	1039	0.294	[0.266; 0.322]	2.5%
Jussupow 2022	Unknown	Unknown	Unknown	126	230	0.548	[0.481; 0.613]	2.5%
Liu 2022	Unknown	Unknown	USA	334	388	0.861	[0.822; 0.894]	2.5%
Lugito 2024	Students	Unknown	Indonesia	9	650	0.014	[0.006; 0.026]	2.3%
Malesivc 2024	Students	Unknown	East Europe	395	1701	0.232	[0.212; 0.253]	2.5%
Pecqueux 2022	Unknown	Unknown	Germany	60	144	0.417	[0.335; 0.502]	2.5%
Pinto 2019	Unknown	Unknown	Germany	126	263	0.479	[0.417; 0.541]	2.5%
Pucchio 2022	Unknown	Unknown	Canada	304	486	0.626	[0.581; 0.669]	2.5%
Robleto 2024	Students	Unknown	USA	40	72	0.556	[0.434; 0.673]	2.4%
Scheetz 2021	Unknown	Unknown	Australia	175	632	0.277	[0.242; 0.314]	2.5%
Shiang 2022	Unknown	Unknown	Isreal	4	15	0.267	[0.078; 0.551]	1.9%
Tangadulrat 2023	Phy+Stu	Unknown	Thailand	51	239	0.213	[0.163; 0.271]	2.5%
Valikodath 2021	Unknown	Unknown	USA	7	80	0.087	[0.036; 0.172]	2.2%
**Random effects model**					**14606**	**0.343**	**[0.244; 0.458]**	**58.2%**
**Prediction interval**							**[0.041; 0.864]**	
Heterogeneity: $I^2 = 99.2\%$, $\tau^2 = 1.3984$, $\chi^2_{23} = 2885.35$ ($p < 0.001$)								
**Specialty = Mixed**								
Al Medfa 2023	Physicians	Mixed	Bahrain	30	166	0.181	[0.125; 0.248]	2.4%
Iyengar 2023	Physicians	Mixed	Europe + Asia	66	125	0.528	[0.437; 0.618]	2.5%
Nishan 2024	Physicians	Mixed	Bangladesh	115	398	0.289	[0.245; 0.336]	2.5%
Orlova 2023	Physicians	Mixed	Russia	151	301	0.502	[0.444; 0.560]	2.5%
Radhwi 2024	Physicians	Mixed	Saudi Arabia	76	128	0.594	[0.503; 0.680]	2.5%
**Random effects model**					**1118**	**0.406**	**[0.269; 0.559]**	**12.3%**
**Prediction interval**							**[0.077; 0.848]**	
Heterogeneity: $I^2 = 95.6\%$, $\tau^2 = 0.4722$, $\chi^2_4 = 90.2$ ($p < 0.001$)								
**Specialty = Family/Internal medicine**								
Alexandrou 2024	Physicians	Family/Internal medicine	North America + Europe	79	521	0.152	[0.122; 0.185]	2.5%
Hantel 2024	Physicians	Family/Internal medicine	USA	92	203	0.453	[0.383; 0.524]	2.5%
McGenity 2024	Physicians	Family/Internal medicine	UK	29	42	0.690	[0.529; 0.824]	2.3%
Sahin 2023	Physicians	Family/Internal medicine	Turkey	101	156	0.647	[0.567; 0.722]	2.5%
Savoia 2023	Physicians	Family/Internal medicine	Europe	158	239	0.661	[0.597; 0.721]	2.5%
Waheed 2024	Physicians	Family/Internal medicine	Qatar	22	108	0.204	[0.132; 0.292]	2.4%
**Random effects model**					**1269**	**0.449**	**[0.243; 0.674]**	**14.6%**
**Prediction interval**							**[0.033; 0.952]**	
Heterogeneity: $I^2 = 98\%$, $\tau^2 = 1.3112$, $\chi^2_5 = 245.51$ ($p < 0.001$)								
**Specialty = Anesthesiology**								
Cai 2024	Physicians	Anesthesiology	China	87	396	0.220	[0.180; 0.264]	2.5%
Estrada Alamo 2024	Physicians	Anesthesiology	USA	418	1083	0.386	[0.357; 0.416]	2.5%
**Random effects model**					**1479**	**0.298**	**[0.162; 0.482]**	**5.0%**
**Prediction interval**							**[0.000; 1.000]**	
Heterogeneity: $I^2 = 97.1\%$, $\tau^2 = 0.3132$, $\chi^2_1 = 34.63$ ($p < 0.001$)								
**Specialty = Emergency medicine**								
Eastwood 2023	Physicians	Emergency medicine	Canada	52	171	0.304	[0.236; 0.379]	2.5%
**Specialty = Radiology**								
Eltorai 2024	Physicians	Radiology	USA	101	211	0.479	[0.410; 0.548]	2.5%
Zanardo 2024	Physicians	Radiology	Europe	153	572	0.267	[0.232; 0.306]	2.5%
**Random effects model**					**783**	**0.365**	**[0.189; 0.587]**	**5.0%**
**Prediction interval**							**[0.000; 1.000]**	
Heterogeneity: $I^2 = 96.7\%$, $\tau^2 = 0.4112$, $\chi^2_1 = 30.46$ ($p < 0.001$)								
**Specialty = Orthodontics**								
Mengi 2024	Phy+Stu	Orthodontics	India	30	100	0.300	[0.212; 0.400]	2.4%
**Random effects model**					**19526**	**0.363**	**[0.293; 0.439]**	**100.0%**
**Prediction interval**							**[0.065; 0.822]**	

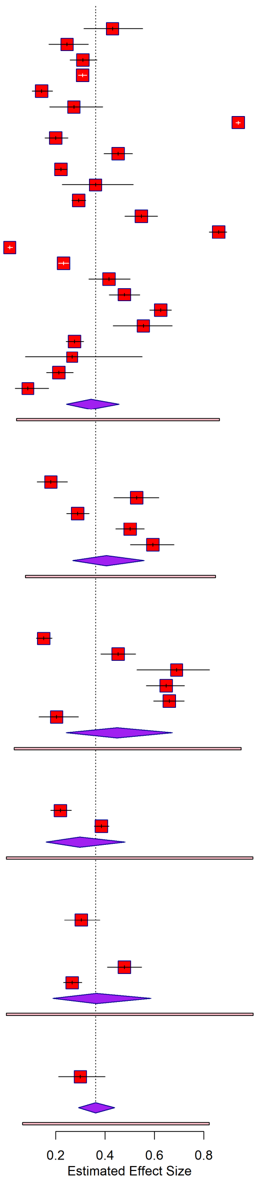

Heterogeneity: $I^2 = 98.8\%$, $\tau^2 = 1.0473$, $\chi^2_{40} = 3312.42$ ($p < 0.001$)
Test for subgroup differences: $\chi^2_6 = 3.43$, df = 6 ($p = 0.75$)

Figure S3. Rates Of **Low AI Knowledge** Across the Included Studies, Stratified by the **Specialty** of the Participants. Each study's effect size represents the percentage of participants with low AI knowledge in that study. The dotted line indicates the overall estimated rate of low AI knowledge.

Study	Participants	Specialty	Country	Case N	Total N	Effect Size	95%CI	w.random
**Participant = Unknown**								
Al-Ali 2023	Unknown	Unknown	Saudi Arabia	33	72	0.458	[0.340; 0.580]	3.9%
Al-Medfa 2023	Unknown	Unknown	Bahrain	76	122	0.623	[0.531; 0.709]	4.1%
Bisdas 2021	Unknown	Unknown	International	1340	3033	0.442	[0.424; 0.460]	4.5%
Eschert 2022	Unknown	Unknown	Germany	146	302	0.483	[0.426; 0.541]	4.4%
Gunasekeran 2022	Unknown	Unknown	International	639	1107	0.577	[0.548; 0.607]	4.5%
Hogberg 2023	Unknown	Unknown	Sweden	26	47	0.553	[0.401; 0.698]	3.7%
Huisman 2021	Unknown	Unknown	Europe	296	1039	0.285	[0.258; 0.313]	4.5%
Pecqueux 2022	Unknown	Unknown	Germany	67	144	0.465	[0.382; 0.550]	4.2%
Scheetz 2021	Unknown	Unknown	Australia	301	632	0.476	[0.437; 0.516]	4.5%
Shiang 2022	Unknown	Unknown	Isreal	9	15	0.600	[0.323; 0.837]	2.5%
**Random effects model**					**6513**	**0.485**	**[0.415; 0.555]**	**40.8%**
**Prediction interval**							**[0.257; 0.719]**	
Heterogeneity: I^2 = 95.5%, τ^2 = 0.1745, χ^2_9 = 201.84 (p < 0.001)								
**Participant = Physicians**								
Al Medfa 2023	Physicians	Mixed	Bahrain	60	166	0.361	[0.288; 0.440]	4.2%
Alexandrou 2024	Physicians	Family/Internal medicine	North America + Europe	242	521	0.464	[0.421; 0.508]	4.4%
Eastwood 2023	Physicians	Emergency medicine	Canada	78	171	0.456	[0.380; 0.534]	4.3%
Estrada Alamo 2024	Physicians	Anesthesiology	USA	588	1083	0.543	[0.513; 0.573]	4.5%
McGenity 2024	Physicians	Family/Internal medicine	UK	7	42	0.167	[0.070; 0.314]	3.1%
Orlova 2023	Physicians	Mixed	Russia	136	301	0.452	[0.395; 0.510]	4.4%
Sahin 2023	Physicians	Family/Internal medicine	Turkey	47	156	0.301	[0.231; 0.380]	4.2%
Savoia 2023	Physicians	Family/Internal medicine	Europe	68	239	0.285	[0.228; 0.346]	4.3%
Waheed 2024	Physicians	Family/Internal medicine	Qatar	55	108	0.509	[0.411; 0.607]	4.1%
Zanardo 2024	Physicians	Radiology	Europe	145	572	0.253	[0.218; 0.291]	4.4%
**Random effects model**					**3359**	**0.380**	**[0.305; 0.461]**	**41.9%**
**Prediction interval**							**[0.153; 0.675]**	
Heterogeneity: I^2 = 94.8%, τ^2 = 0.2626, χ^2_9 = 174.32 (p < 0.001)								
**Participant = Students**								
Lugito 2024	Students	Unknown	Indonesia	528	650	0.812	[0.780; 0.842]	4.4%
Malesivc 2024	Students	Unknown	East Europe	1148	1701	0.675	[0.652; 0.697]	4.5%
**Random effects model**					**2351**	**0.749**	**[0.592; 0.860]**	**8.9%**
**Prediction interval**							**[0.001; 1.000]**	
Heterogeneity: I^2 = 97.6%, τ^2 = 0.2635, χ^2_1 = 42.26 (p < 0.001)								
**Participant = Phy+Stu**								
Mengi 2024	Phy+Stu	Orthodontics	India	66	100	0.660	[0.558; 0.752]	4.0%
Tangadulrat 2023	Phy+Stu	Unknown	Thailand	150	239	0.628	[0.563; 0.689]	4.3%
**Random effects model**					**339**	**0.637**	**[0.584; 0.687]**	**8.4%**
**Prediction interval**							**[0.295; 0.881]**	
Heterogeneity: I^2 = 0%, τ^2 = 0, χ^2_1 = 0.32 (p = 0.57)								
**Random effects model**					**12562**	**0.480**	**[0.418; 0.542]**	**100.0%**
**Prediction interval**							**[0.207; 0.765]**	

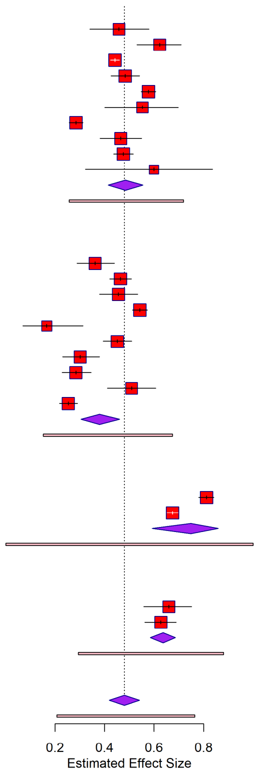

Heterogeneity: I^2 = 97.5%, τ^2 = 0.3546, χ^2_{23} = 926.96 (p < 0.001)
Test for subgroup differences: χ^2_3 = 35.83, df = 3 (p < 0.001)

Figure S4. Rates Of **Medium AI Knowledge** Across the Included Studies, Stratified by the **Type** of the Participants. Each study's effect size represents the percentage of participants with low AI knowledge in that study. The dotted line indicates the overall estimated rate of medium AI knowledge.

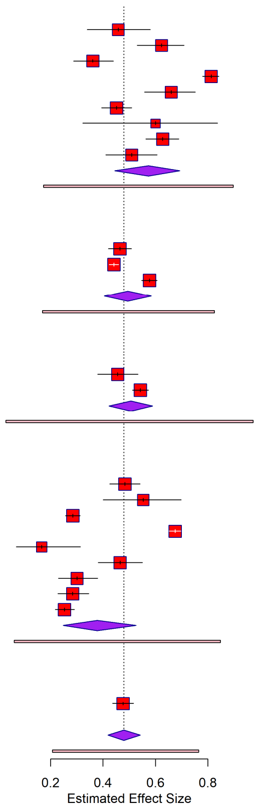

Study	Participants	Specialty	Country	Case N	Total N	Effect Size	95%CI	w.random
**Country = Asia**								
Al-Ali 2023	Unknown	Unknown	Saudi Arabia	33	72	0.458	[0.340; 0.580]	3.9%
Al-Medfa 2023	Unknown	Unknown	Bahrain	76	122	0.623	[0.531; 0.709]	4.1%
Al Medfa 2023	Physicians	Mixed	Bahrain	60	166	0.361	[0.288; 0.440]	4.2%
Lugito 2024	Students	Unknown	Indonesia	528	650	0.812	[0.780; 0.842]	4.4%
Mengi 2024	Phy+Stu	Orthodontics	India	66	100	0.660	[0.558; 0.752]	4.0%
Orlova 2023	Physicians	Mixed	Russia	136	301	0.452	[0.395; 0.510]	4.4%
Shiang 2022	Unknown	Unknown	Isreal	9	15	0.600	[0.323; 0.837]	2.5%
Tangadulrat 2023	Phy+Stu	Unknown	Thailand	150	239	0.628	[0.563; 0.689]	4.3%
Waheed 2024	Physicians	Family/Internal medicine	Qatar	55	108	0.509	[0.411; 0.607]	4.1%
**Random effects model**					**1773**	**0.574**	**[0.445; 0.693]**	**36.1%**
**Prediction interval**							**[0.173; 0.896]**	
Heterogeneity: I^2 = 95.7%, τ^2 = 0.5792, χ^2_8 = 185.55 (p < 0.001)								
**Country = International**								
Alexandrou 2024	Physicians	Family/Internal medicine	North America + Europe	242	521	0.464	[0.421; 0.508]	4.4%
Bisdas 2021	Unknown	Unknown	International	1340	3033	0.442	[0.424; 0.460]	4.5%
Gunasekeran 2022	Unknown	Unknown	International	639	1107	0.577	[0.548; 0.607]	4.5%
**Random effects model**					**4661**	**0.495**	**[0.405; 0.584]**	**13.5%**
**Prediction interval**							**[0.169; 0.824]**	
Heterogeneity: I^2 = 96.6%, τ^2 = 0.0985, χ^2_2 = 59.33 (p < 0.001)								
**Country = N America**								
Eastwood 2023	Physicians	Emergency medicine	Canada	78	171	0.456	[0.380; 0.534]	4.3%
Estrada Alamo 2024	Physicians	Anesthesiology	USA	588	1083	0.543	[0.513; 0.573]	4.5%
**Random effects model**					**1254**	**0.507**	**[0.423; 0.590]**	**8.8%**
**Prediction interval**							**[0.030; 0.972]**	
Heterogeneity: I^2 = 77.5%, τ^2 = 0.0469, χ^2_1 = 4.44 (p = 0.04)								
**Country = Europe**								
Eschert 2022	Unknown	Unknown	Germany	146	302	0.483	[0.426; 0.541]	4.4%
Hogberg 2023	Unknown	Unknown	Sweden	26	47	0.553	[0.401; 0.698]	3.7%
Huisman 2021	Unknown	Unknown	Europe	296	1039	0.285	[0.258; 0.313]	4.5%
Malesivc 2024	Students	Unknown	East Europe	1148	1701	0.675	[0.652; 0.697]	4.5%
McGenity 2024	Physicians	Family/Internal medicine	UK	7	42	0.167	[0.070; 0.314]	3.1%
Pecqueux 2022	Unknown	Unknown	Germany	67	144	0.465	[0.382; 0.550]	4.2%
Sahin 2023	Physicians	Family/Internal medicine	Turkey	47	156	0.301	[0.231; 0.380]	4.2%
Savoia 2023	Physicians	Family/Internal medicine	Europe	68	239	0.285	[0.228; 0.346]	4.3%
Zanardo 2024	Physicians	Radiology	Europe	145	572	0.253	[0.218; 0.291]	4.4%
**Random effects model**					**4242**	**0.377**	**[0.248; 0.527]**	**37.2%**
**Prediction interval**							**[0.062; 0.848]**	
Heterogeneity: I^2 = 98.6%, τ^2 = 0.8294, χ^2_8 = 567.67 (p < 0.001)								
**Country = Pacific**								
Scheetz 2021	Unknown	Unknown	Australia	301	632	0.476	[0.437; 0.516]	4.5%
**Random effects model**					**12562**	**0.480**	**[0.418; 0.542]**	**100.0%**
**Prediction interval**							**[0.207; 0.765]**	

Heterogeneity: I^2 = 97.5%, τ^2 = 0.3546, χ^2_{23} = 926.96 (p < 0.001)
Test for subgroup differences: χ^2_4 = 4.32, df = 4 (p = 0.36)

Figure S5. Rates Of **Medium AI Knowledge** Across the Included Studies, Stratified by the **Country** of the Participants. Each study's effect size represents the percentage of participants with medium AI knowledge in that study. The dotted line indicates the overall estimated rate of medium AI knowledge.

Study	Participants	Specialty	Country	Case N	Total N	Effect Size	95%CI	w.random
**Specialty = Unknown**								
Al-Ali 2023	Unknown	Unknown	Saudi Arabia	33	72	0.458	[0.340; 0.580]	3.9%
Al-Medfa 2023	Unknown	Unknown	Bahrain	76	122	0.623	[0.531; 0.709]	4.1%
Bisdas 2021	Unknown	Unknown	International	1340	3033	0.442	[0.424; 0.460]	4.5%
Eschert 2022	Unknown	Unknown	Germany	146	302	0.483	[0.426; 0.541]	4.4%
Gunasekeran 2022	Unknown	Unknown	International	639	1107	0.577	[0.548; 0.607]	4.5%
Hogberg 2023	Unknown	Unknown	Sweden	26	47	0.553	[0.401; 0.698]	3.7%
Huisman 2021	Unknown	Unknown	Europe	296	1039	0.285	[0.258; 0.313]	4.5%
Lugito 2024	Students	Unknown	Indonesia	528	650	0.812	[0.780; 0.842]	4.4%
Malesivc 2024	Students	Unknown	East Europe	1148	1701	0.675	[0.652; 0.697]	4.5%
Pecqueux 2022	Unknown	Unknown	Germany	67	144	0.465	[0.382; 0.550]	4.2%
Scheetz 2021	Unknown	Unknown	Australia	301	632	0.476	[0.437; 0.516]	4.5%
Shiang 2022	Unknown	Unknown	Isreal	9	15	0.600	[0.323; 0.837]	2.5%
Tangadulrat 2023	Phy+Stu	Unknown	Thailand	150	239	0.628	[0.563; 0.689]	4.3%
**Random effects model**					**9103**	**0.547**	**[0.458; 0.633]**	**54.1%**
**Prediction interval**							**[0.225; 0.834]**	
Heterogeneity: $I^2 = 98.2\%$, $\tau^2 = 0.3949$, $\chi^2_{12} = 673.71$ ($p < 0.001$)								
**Specialty = Mixed**								
Al Medfa 2023	Physicians	Mixed	Bahrain	60	166	0.361	[0.288; 0.440]	4.2%
Orlova 2023	Physicians	Mixed	Russia	136	301	0.452	[0.395; 0.510]	4.4%
**Random effects model**					**467**	**0.410**	**[0.325; 0.501]**	**8.6%**
**Prediction interval**							**[0.016; 0.966]**	
Heterogeneity: $I^2 = 72\%$, $\tau^2 = 0.0509$, $\chi^2_1 = 3.57$ ($p = 0.06$)								
**Specialty = Family/Internal medicine**								
Alexandrou 2024	Physicians	Family/Internal medicine	North America + Europe	242	521	0.464	[0.421; 0.508]	4.4%
McGenity 2024	Physicians	Family/Internal medicine	UK	7	42	0.167	[0.070; 0.314]	3.1%
Sahin 2023	Physicians	Family/Internal medicine	Turkey	47	156	0.301	[0.231; 0.380]	4.2%
Savoia 2023	Physicians	Family/Internal medicine	Europe	68	239	0.285	[0.228; 0.346]	4.3%
Waheed 2024	Physicians	Family/Internal medicine	Qatar	55	108	0.509	[0.411; 0.607]	4.1%
**Random effects model**					**1066**	**0.349**	**[0.251; 0.461]**	**20.1%**
**Prediction interval**							**[0.105; 0.709]**	
Heterogeneity: $I^2 = 90.5\%$, $\tau^2 = 0.2404$, $\chi^2_4 = 41.94$ ($p < 0.001$)								
**Specialty = Emergency medicine**								
Eastwood 2023	Physicians	Emergency medicine	Canada	78	171	0.456	[0.380; 0.534]	4.3%
**Specialty = Anesthesiology**								
Estrada Alamo 2024	Physicians	Anesthesiology	USA	588	1083	0.543	[0.513; 0.573]	4.5%
**Specialty = Orthodontics**								
Mengi 2024	Phy+Stu	Orthodontics	India	66	100	0.660	[0.558; 0.752]	4.0%
**Specialty = Radiology**								
Zanardo 2024	Physicians	Radiology	Europe	145	572	0.253	[0.218; 0.291]	4.4%
**Random effects model**					**12562**	**0.480**	**[0.418; 0.542]**	**100.0%**
**Prediction interval**							**[0.207; 0.765]**	

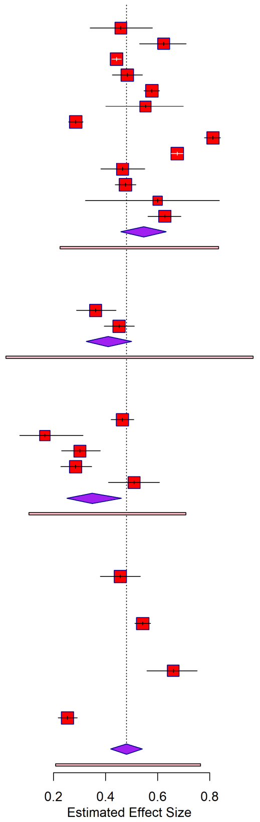

Heterogeneity: $I^2 = 97.5\%$, $\tau^2 = 0.3546$, $\chi^2_{23} = 926.96$ ($p < 0.001$)
Test for subgroup differences: $\chi^2_6 = 145.15$, df = 6 ($p < 0.001$)

Figure S6. Rates Of **Medium AI Knowledge** Across the Included Studies, Stratified by the **Specialty** of the Participants. Each study's effect size represents the percentage of participants with medium AI knowledge in that study. The dotted line indicates the overall estimated rate of medium AI knowledge.

Study	Participants	Specialty	Country	Case N	Total N	Effect Size	95%CI	w.random
**Participant = Unknown**								
Ahmed 2022	Unknown	Unknown	Qatar	71	202	0.351	[0.286; 0.422]	3.7%
Al-Ali 2023	Unknown	Unknown	Saudi Arabia	8	72	0.111	[0.049; 0.207]	3.1%
Al-Medfa 2023	Unknown	Unknown	Bahrain	16	122	0.131	[0.077; 0.204]	3.5%
Bisdas 2021	Unknown	Unknown	International	755	3033	0.249	[0.234; 0.265]	3.9%
Eschert 2022	Unknown	Unknown	Germany	19	302	0.063	[0.038; 0.097]	3.5%
Gunasekeran 2022	Unknown	Unknown	International	226	1107	0.204	[0.181; 0.229]	3.8%
Hogberg 2023	Unknown	Unknown	Sweden	4	47	0.085	[0.024; 0.204]	2.7%
Huisman 2021	Unknown	Unknown	Europe	168	1039	0.162	[0.140; 0.186]	3.8%
Leenhardt 2021	Unknown	Unknown	International	151	380	0.397	[0.348; 0.449]	3.8%
Lim 2022	Unknown	Unknown	Australia	58	88	0.659	[0.550; 0.757]	3.6%
Pecqueux 2022	Unknown	Unknown	Germany	17	144	0.118	[0.070; 0.182]	3.5%
Scheetz 2021	Unknown	Unknown	Australia	156	632	0.247	[0.214; 0.282]	3.8%
Shiang 2022	Unknown	Unknown	Isreal	2	15	0.133	[0.017; 0.405]	2.0%
**Random effects model**					**7183**	**0.210**	**[0.163; 0.265]**	**44.7%**
**Prediction interval**							**[0.076; 0.459]**	
Heterogeneity: $I^2 = 95.1\%$, $\tau^2 = 0.2603$, $\chi^2_{12} = 244.85$ ($p < 0.001$)								
**Participant = Physicians**								
Al Harbi 2024	Physicians	Anesthesiology	Saudi Arabia	215	466	0.461	[0.415; 0.508]	3.8%
Al Medfa 2023	Physicians	Mixed	Bahrain	76	166	0.458	[0.380; 0.537]	3.7%
Alexandrou 2024	Physicians	Family/Internal medicine	North America + Europe	200	521	0.384	[0.342; 0.427]	3.8%
Eastwood 2023	Physicians	Emergency medicine	Canada	40	171	0.234	[0.173; 0.305]	3.7%
Estrada Alamo 2024	Physicians	Anesthesiology	USA	77	1083	0.071	[0.057; 0.088]	3.8%
Henckert 2023	Physicians	Anesthesiology	Europe	47	49	0.959	[0.860; 0.995]	2.1%
McGenity 2024	Physicians	Family/Internal medicine	UK	6	42	0.143	[0.054; 0.285]	2.9%
Orlova 2023	Physicians	Mixed	Russia	14	301	0.047	[0.026; 0.077]	3.4%
Sahin 2023	Physicians	Family/Internal medicine	Turkey	8	156	0.051	[0.022; 0.099]	3.2%
Savoia 2023	Physicians	Family/Internal medicine	Europe	13	239	0.054	[0.029; 0.091]	3.4%
Waheed 2024	Physicians	Family/Internal medicine	Qatar	22	108	0.204	[0.132; 0.292]	3.5%
Zanardo 2024	Physicians	Radiology	Europe	274	572	0.479	[0.437; 0.521]	3.8%
**Random effects model**					**3874**	**0.233**	**[0.139; 0.363]**	**41.2%**
**Prediction interval**							**[0.025; 0.782]**	
Heterogeneity: $I^2 = 98.1\%$, $\tau^2 = 1.1557$, $\chi^2_{11} = 570.85$ ($p < 0.001$)								
**Participant = Students**								
Lugito 2024	Students	Unknown	Indonesia	113	650	0.174	[0.145; 0.205]	3.8%
Malesivc 2024	Students	Unknown	East Europe	160	1701	0.094	[0.081; 0.109]	3.8%
**Random effects model**					**2351**	**0.128**	**[0.069; 0.228]**	**7.6%**
**Prediction interval**							**[0.000; 0.997]**	
Heterogeneity: $I^2 = 96.5\%$, $\tau^2 = 0.2407$, $\chi^2_1 = 28.34$ ($p < 0.001$)								
**Participant = Phy+Stu**								
Mengi 2024	Phy+Stu	Orthodontics	India	4	100	0.040	[0.011; 0.099]	2.7%
Tangadulrat 2023	Phy+Stu	Unknown	Thailand	38	239	0.159	[0.115; 0.212]	3.7%
**Random effects model**					**339**	**0.087**	**[0.021; 0.295]**	**6.4%**
**Prediction interval**							**[0.000; 1.000]**	
Heterogeneity: $I^2 = 87.2\%$, $\tau^2 = 0.9977$, $\chi^2_1 = 7.84$ ($p = 0.005$)								
**Random effects model**					**13747**	**0.196**	**[0.153; 0.248]**	**100.0%**
**Prediction interval**							**[0.046; 0.550]**	

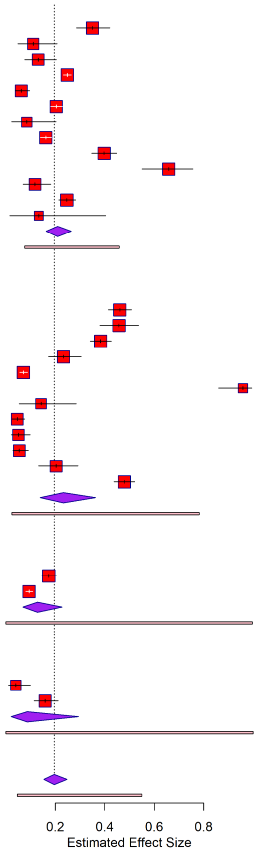

Heterogeneity: $I^2 = 97.5\%$, $\tau^2 = 0.5960$, $\chi^2_{28} = 1127.02$ ($p < 0.001$)
Test for subgroup differences: $\chi^2_3 = 4.32$, df = 3 ($p = 0.23$)

Figure S7. Rates Of **<u>High AI Knowledge</u>** Across the Included Studies, Stratified by the **<u>Type</u>** of the Participants. Each study's effect size represents the percentage of participants with high AI knowledge in that study. The dotted line indicates the overall estimated rate of high AI knowledge.

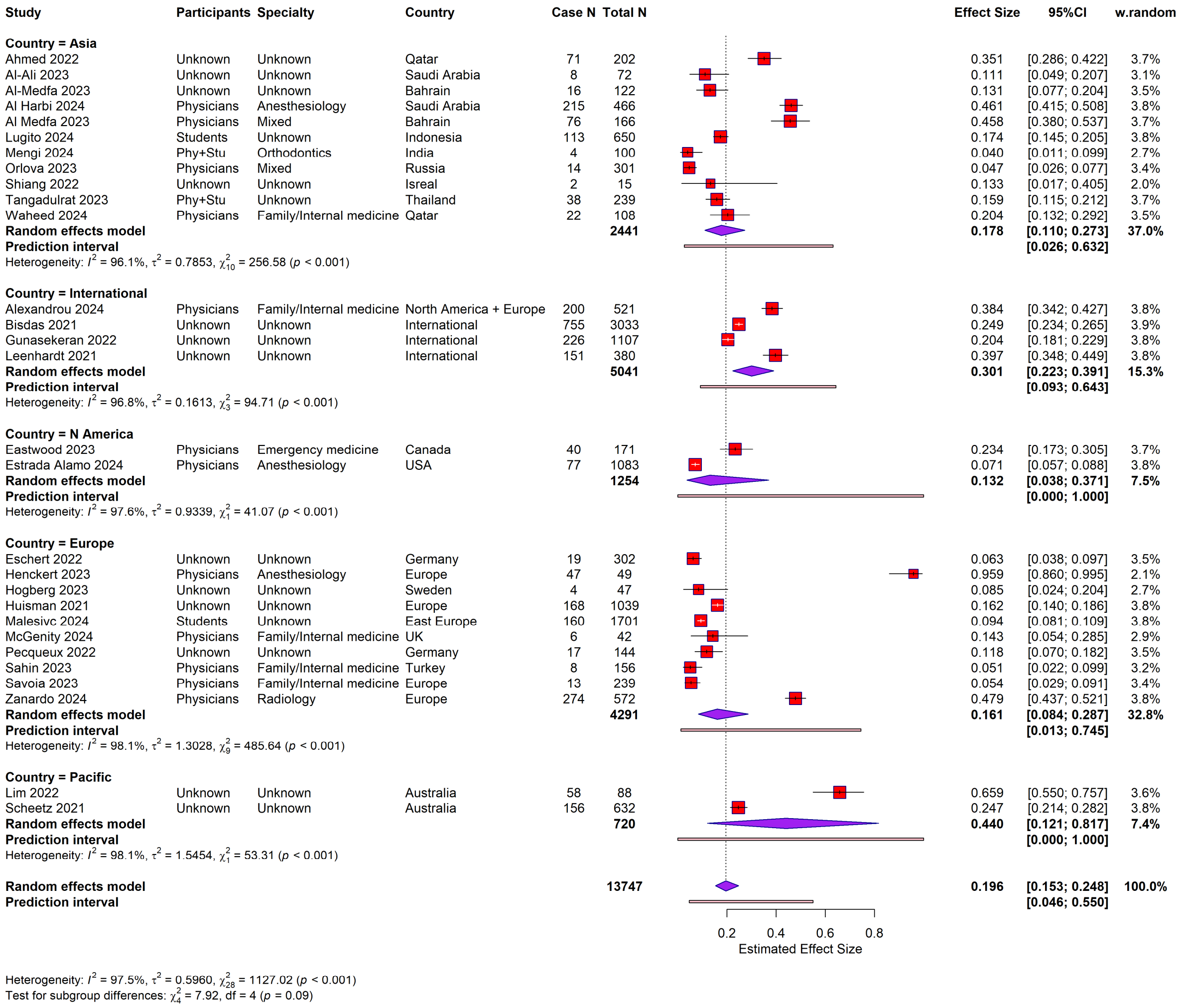

Figure S8. Rates Of **High AI Knowledge** Across the Included Studies, Stratified by the **Country** of the Participants. Each study's effect size represents the percentage of participants with high AI knowledge in that study. The dotted line indicates the overall estimated rate of high AI knowledge.

Study	Participants	Specialty	Country	Case N	Total N	Effect Size	95%CI	w.random
**Speciatly = Unknown**								
Ahmed 2022	Unknown	Unknown	Qatar	71	202	0.351	[0.286; 0.422]	3.7%
Al-Ali 2023	Unknown	Unknown	Saudi Arabia	8	72	0.111	[0.049; 0.207]	3.1%
Al-Medfa 2023	Unknown	Unknown	Bahrain	16	122	0.131	[0.077; 0.204]	3.5%
Bisdas 2021	Unknown	Unknown	International	755	3033	0.249	[0.234; 0.265]	3.9%
Eschert 2022	Unknown	Unknown	Germany	19	302	0.063	[0.038; 0.097]	3.5%
Gunasekeran 2022	Unknown	Unknown	International	226	1107	0.204	[0.181; 0.229]	3.8%
Hogberg 2023	Unknown	Unknown	Sweden	4	47	0.085	[0.024; 0.204]	2.7%
Huisman 2021	Unknown	Unknown	Europe	168	1039	0.162	[0.140; 0.186]	3.8%
Leenhardt 2021	Unknown	Unknown	International	151	380	0.397	[0.348; 0.449]	3.8%
Lim 2022	Unknown	Unknown	Australia	58	88	0.659	[0.550; 0.757]	3.6%
Lugito 2024	Students	Unknown	Indonesia	113	650	0.174	[0.145; 0.205]	3.8%
Malesivc 2024	Students	Unknown	East Europe	160	1701	0.094	[0.081; 0.109]	3.8%
Pecqueux 2022	Unknown	Unknown	Germany	17	144	0.118	[0.070; 0.182]	3.5%
Scheetz 2021	Unknown	Unknown	Australia	156	632	0.247	[0.214; 0.282]	3.8%
Shiang 2022	Unknown	Unknown	Isreal	2	15	0.133	[0.017; 0.405]	2.0%
Tangadulrat 2023	Phy+Stu	Unknown	Thailand	38	239	0.159	[0.115; 0.212]	3.7%
**Random effects model**					**9773**	**0.191**	**[0.148; 0.242]**	**56.1%**
**Prediction interval**							**[0.062; 0.457]**	
Heterogeneity: $I^2 = 96.4\%$, $\tau^2 = 0.3315$, $\chi^2_{15} = 413.87$ ($p < 0.001$)								
**Speciatly = Anesthesiology**								
Al Harbi 2024	Physicians	Anesthesiology	Saudi Arabia	215	466	0.461	[0.415; 0.508]	3.8%
Estrada Alamo 2024	Physicians	Anesthesiology	USA	77	1083	0.071	[0.057; 0.088]	3.8%
Henckert 2023	Physicians	Anesthesiology	Europe	47	49	0.959	[0.860; 0.995]	2.1%
**Random effects model**					**1598**	**0.500**	**[0.112; 0.888]**	**9.7%**
**Prediction interval**							**[0.000; 1.000]**	
Heterogeneity: $I^2 = 99.3\%$, $\tau^2 = 3.1746$, $\chi^2_{2} = 291.94$ ($p < 0.001$)								
**Speciatly = Mixed**								
Al Medfa 2023	Physicians	Mixed	Bahrain	76	166	0.458	[0.380; 0.537]	3.7%
Orlova 2023	Physicians	Mixed	Russia	14	301	0.047	[0.026; 0.077]	3.4%
**Random effects model**					**467**	**0.170**	**[0.012; 0.770]**	**7.2%**
**Prediction interval**							**[0.000; 1.000]**	
Heterogeneity: $I^2 = 98.8\%$, $\tau^2 = 4.0155$, $\chi^2_{1} = 81.97$ ($p < 0.001$)								
**Speciatly = Family/Internal medicine**								
Alexandrou 2024	Physicians	Family/Internal medicine	North America + Europe	200	521	0.384	[0.342; 0.427]	3.8%
McGenity 2024	Physicians	Family/Internal medicine	UK	6	42	0.143	[0.054; 0.285]	2.9%
Sahin 2023	Physicians	Family/Internal medicine	Turkey	8	156	0.051	[0.022; 0.099]	3.2%
Savoia 2023	Physicians	Family/Internal medicine	Europe	13	239	0.054	[0.029; 0.091]	3.4%
Waheed 2024	Physicians	Family/Internal medicine	Qatar	22	108	0.204	[0.132; 0.292]	3.5%
**Random effects model**					**1066**	**0.135**	**[0.050; 0.318]**	**16.9%**
**Prediction interval**							**[0.004; 0.864]**	
Heterogeneity: $I^2 = 96.3\%$, $\tau^2 = 1.4699$, $\chi^2_{4} = 108.22$ ($p < 0.001$)								
**Speciatly = Emergency medicine**								
Eastwood 2023	Physicians	Emergency medicine	Canada	40	171	0.234	[0.173; 0.305]	3.7%
**Speciatly = Orthodontics**								
Mengi 2024	Phy+Stu	Orthodontics	India	4	100	0.040	[0.011; 0.099]	2.7%
**Speciatly = Radiology**								
Zanardo 2024	Physicians	Radiology	Europe	274	572	0.479	[0.437; 0.521]	3.8%
**Random effects model**					**13747**	**0.196**	**[0.153; 0.248]**	**100.0%**
**Prediction interval**							**[0.046; 0.550]**	

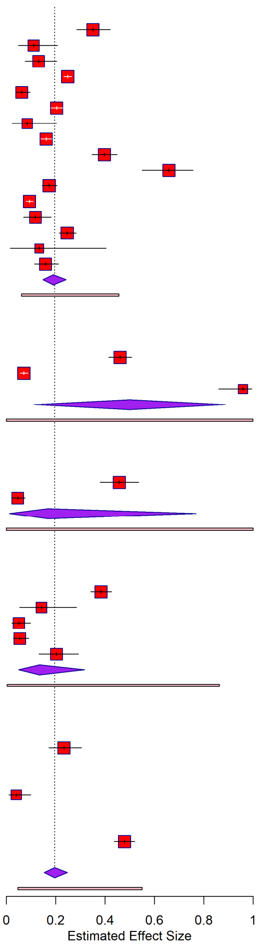

Heterogeneity: $I^2 = 97.5\%$, $\tau^2 = 0.5960$, $\chi^2_{28} = 1127.02$ ($p < 0.001$)
Test for subgroup differences: $\chi^2_{6} = 109.17$, df = 6 ($p < 0.001$)

Figure S9. Rates Of **High AI Knowledge** Across the Included Studies, Stratified by the **Specialty** of the Participants. Each study's effect size represents the percentage of participants with high AI knowledge in that study. The dotted line indicates the overall estimated rate of high AI knowledge.

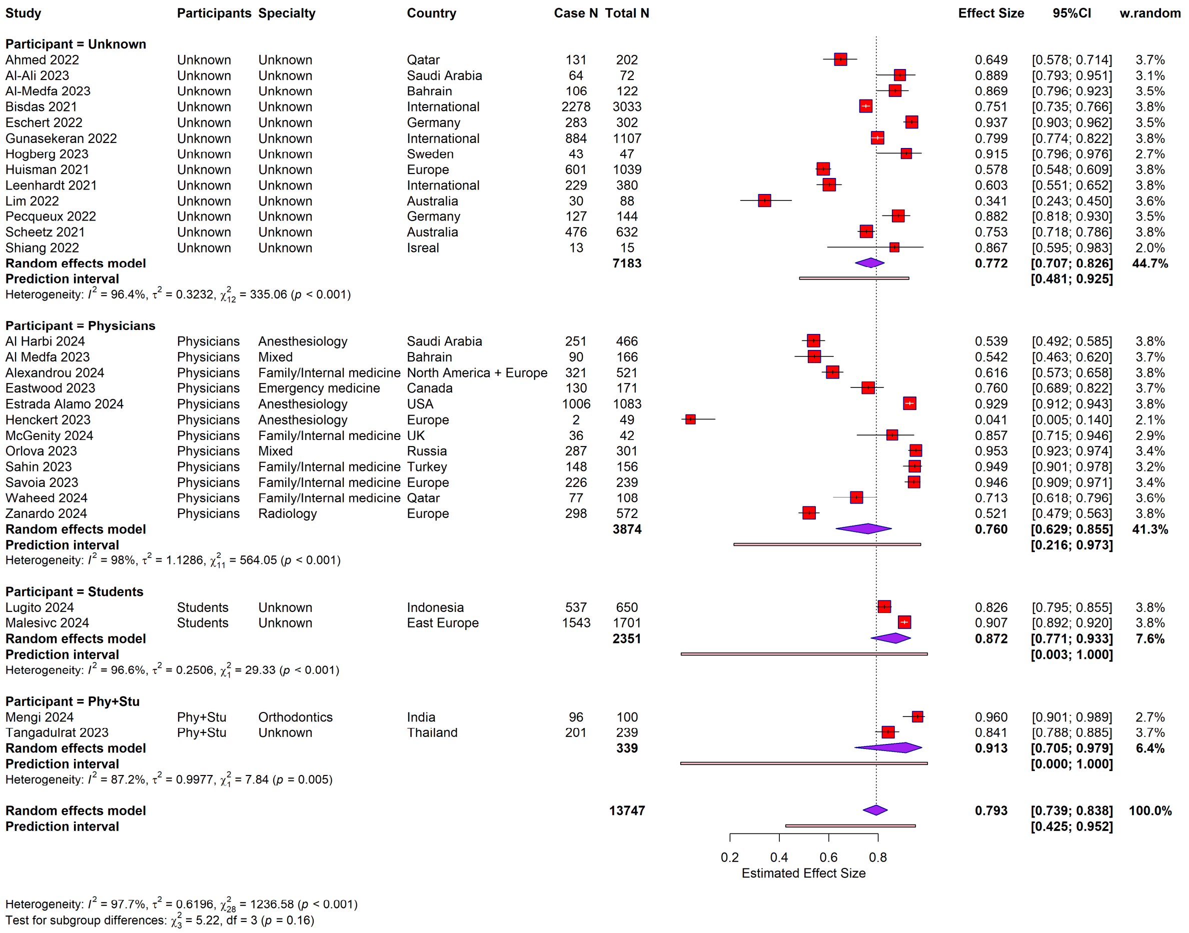

Figure S10. Rates Of **Low or Medium AI Knowledge** Across the Included Studies, Stratified by the **Type** of the Participants. Each study's effect size represents the percentage of participants with low or medium AI knowledge in that study. The dotted line indicates the overall estimated rate of low or medium AI knowledge.

Study	Participants	Specialty	Country	Case N	Total N	Effect Size	95%CI	w.random
**Country = Asia**								
Ahmed 2022	Unknown	Unknown	Qatar	131	202	0.649	[0.578; 0.714]	3.7%
Al-Ali 2023	Unknown	Unknown	Saudi Arabia	64	72	0.889	[0.793; 0.951]	3.1%
Al-Medfa 2023	Unknown	Unknown	Bahrain	106	122	0.869	[0.796; 0.923]	3.5%
Al Harbi 2024	Physicians	Anesthesiology	Saudi Arabia	251	466	0.539	[0.492; 0.585]	3.8%
Al Medfa 2023	Physicians	Mixed	Bahrain	90	166	0.542	[0.463; 0.620]	3.7%
Lugito 2024	Students	Unknown	Indonesia	537	650	0.826	[0.795; 0.855]	3.8%
Mengi 2024	Phy+Stu	Orthodontics	India	96	100	0.960	[0.901; 0.989]	2.7%
Orlova 2023	Physicians	Mixed	Russia	287	301	0.953	[0.923; 0.974]	3.4%
Shiang 2022	Unknown	Unknown	Isreal	13	15	0.867	[0.595; 0.983]	2.0%
Tangadulrat 2023	Phy+Stu	Unknown	Thailand	201	239	0.841	[0.788; 0.885]	3.7%
Waheed 2024	Physicians	Family/Internal medicine	Qatar	77	108	0.713	[0.618; 0.796]	3.6%
**Random effects model**					**2441**	**0.816**	**[0.719; 0.884]**	**37.1%**
**Prediction interval**							**[0.364; 0.972]**	
Heterogeneity: I^2 = 96.1%, τ^2 = 0.7641, χ^2_{10} = 253.71 (p < 0.001)								
**Country = International**								
Alexandrou 2024	Physicians	Family/Internal medicine	North America + Europe	321	521	0.616	[0.573; 0.658]	3.8%
Bisdas 2021	Unknown	Unknown	International	2278	3033	0.751	[0.735; 0.766]	3.8%
Gunasekeran 2022	Unknown	Unknown	International	884	1107	0.799	[0.774; 0.822]	3.8%
Leenhardt 2021	Unknown	Unknown	International	229	380	0.603	[0.551; 0.652]	3.8%
**Random effects model**					**5041**	**0.700**	**[0.609; 0.778]**	**15.3%**
**Prediction interval**							**[0.354; 0.909]**	
Heterogeneity: I^2 = 96.9%, τ^2 = 0.1653, χ^2_3 = 96.64 (p < 0.001)								
**Country = N America**								
Eastwood 2023	Physicians	Emergency medicine	Canada	130	171	0.760	[0.689; 0.822]	3.7%
Estrada Alamo 2024	Physicians	Anesthesiology	USA	1006	1083	0.929	[0.912; 0.943]	3.8%
**Random effects model**					**1254**	**0.866**	**[0.618; 0.963]**	**7.4%**
**Prediction interval**							**[0.000; 1.000]**	
Heterogeneity: I^2 = 97.7%, τ^2 = 0.9795, χ^2_1 = 43.53 (p < 0.001)								
**Country = Europe**								
Eschert 2022	Unknown	Unknown	Germany	283	302	0.937	[0.903; 0.962]	3.5%
Henckert 2023	Physicians	Anesthesiology	Europe	2	49	0.041	[0.005; 0.140]	2.1%
Hogberg 2023	Unknown	Unknown	Sweden	43	47	0.915	[0.796; 0.976]	2.7%
Huisman 2021	Unknown	Unknown	Europe	601	1039	0.578	[0.548; 0.609]	3.8%
Malesivc 2024	Students	Unknown	East Europe	1543	1701	0.907	[0.892; 0.920]	3.8%
McGenity 2024	Physicians	Family/Internal medicine	UK	36	42	0.857	[0.715; 0.946]	2.9%
Pecqueux 2022	Unknown	Unknown	Germany	127	144	0.882	[0.818; 0.930]	3.5%
Sahin 2023	Physicians	Family/Internal medicine	Turkey	148	156	0.949	[0.901; 0.978]	3.2%
Savoia 2023	Physicians	Family/Internal medicine	Europe	226	239	0.946	[0.909; 0.971]	3.4%
Zanardo 2024	Physicians	Radiology	Europe	298	572	0.521	[0.479; 0.563]	3.8%
**Random effects model**					**4291**	**0.817**	**[0.670; 0.908]**	**32.8%**
**Prediction interval**							**[0.194; 0.988]**	
Heterogeneity: I^2 = 98.6%, τ^2 = 1.5024, χ^2_9 = 650.74 (p < 0.001)								
**Country = Pacific**								
Lim 2022	Unknown	Unknown	Australia	30	88	0.341	[0.243; 0.450]	3.6%
Scheetz 2021	Unknown	Unknown	Australia	476	632	0.753	[0.718; 0.786]	3.8%
**Random effects model**					**720**	**0.560**	**[0.183; 0.879]**	**7.4%**
**Prediction interval**							**[0.000; 1.000]**	
Heterogeneity: I^2 = 98.1%, τ^2 = 1.5454, χ^2_1 = 53.31 (p < 0.001)								
**Random effects model**					**13747**	**0.793**	**[0.739; 0.838]**	**100.0%**
**Prediction interval**							**[0.425; 0.952]**	

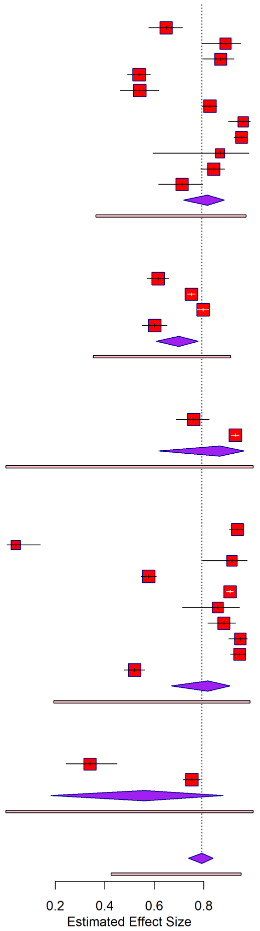

Heterogeneity: I^2 = 97.7%, τ^2 = 0.6196, χ^2_{28} = 1236.58 (p < 0.001)
Test for subgroup differences: χ^2_4 = 6.38, df = 4 (p = 0.17)

Figure S11. Rates Of **Low or Medium AI Knowledge** Across the Included Studies, Stratified by the **Country** of the Participants. Each study's effect size represents the percentage of participants with low or medium AI knowledge in that study. The dotted line indicates the overall estimated rate of low or medium AI knowledge.

Study	Participants	Specialty	Country	Case N	Total N	Effect Size	95%CI	w.random
**Speciatly = Unknown**								
Ahmed 2022	Unknown	Unknown	Qatar	131	202	0.649	[0.578; 0.714]	3.7%
Al-Ali 2023	Unknown	Unknown	Saudi Arabia	64	72	0.889	[0.793; 0.951]	3.1%
Al-Medfa 2023	Unknown	Unknown	Bahrain	106	122	0.869	[0.796; 0.923]	3.5%
Bisdas 2021	Unknown	Unknown	International	2278	3033	0.751	[0.735; 0.766]	3.8%
Eschert 2022	Unknown	Unknown	Germany	283	302	0.937	[0.903; 0.962]	3.5%
Gunasekeran 2022	Unknown	Unknown	International	884	1107	0.799	[0.774; 0.822]	3.8%
Hogberg 2023	Unknown	Unknown	Sweden	43	47	0.915	[0.796; 0.976]	2.7%
Huisman 2021	Unknown	Unknown	Europe	601	1039	0.578	[0.548; 0.609]	3.8%
Leenhardt 2021	Unknown	Unknown	International	229	380	0.603	[0.551; 0.652]	3.8%
Lim 2022	Unknown	Unknown	Australia	30	88	0.341	[0.243; 0.450]	3.6%
Lugito 2024	Students	Unknown	Indonesia	537	650	0.826	[0.795; 0.855]	3.8%
Malesivc 2024	Students	Unknown	East Europe	1543	1701	0.907	[0.892; 0.920]	3.8%
Pecqueux 2022	Unknown	Unknown	Germany	127	144	0.882	[0.818; 0.930]	3.5%
Scheetz 2021	Unknown	Unknown	Australia	476	632	0.753	[0.718; 0.786]	3.8%
Shiang 2022	Unknown	Unknown	Isreal	13	15	0.867	[0.595; 0.983]	2.0%
Tangadulrat 2023	Phy+Stu	Unknown	Thailand	201	239	0.841	[0.788; 0.885]	3.7%
**Random effects model**					**9773**	**0.796**	**[0.734; 0.847]**	**56.0%**
**Prediction interval**							**[0.474; 0.944]**	
Heterogeneity: I^2 = 97.5%, τ^2 = 0.4429, χ^2_{15} = 592.97 (p < 0.001)								
**Speciatly = Anesthesiology**								
Al Harbi 2024	Physicians	Anesthesiology	Saudi Arabia	251	466	0.539	[0.492; 0.585]	3.8%
Estrada Alamo 2024	Physicians	Anesthesiology	USA	1006	1083	0.929	[0.912; 0.943]	3.8%
Henckert 2023	Physicians	Anesthesiology	Europe	2	49	0.041	[0.005; 0.140]	2.1%
**Random effects model**					**1598**	**0.500**	**[0.112; 0.888]**	**9.7%**
**Prediction interval**							**[0.000; 1.000]**	
Heterogeneity: I^2 = 99.3%, τ^2 = 3.1746, χ^2_{2} = 291.94 (p < 0.001)								
**Speciatly = Mixed**								
Al Medfa 2023	Physicians	Mixed	Bahrain	90	166	0.542	[0.463; 0.620]	3.7%
Orlova 2023	Physicians	Mixed	Russia	287	301	0.953	[0.923; 0.974]	3.4%
**Random effects model**					**467**	**0.830**	**[0.230; 0.988]**	**7.2%**
**Prediction interval**							**[0.000; 1.000]**	
Heterogeneity: I^2 = 98.8%, τ^2 = 4.0155, χ^2_{1} = 81.97 (p < 0.001)								
**Speciatly = Family/Internal medicine**								
Alexandrou 2024	Physicians	Family/Internal medicine	North America + Europe	321	521	0.616	[0.573; 0.658]	3.8%
McGenity 2024	Physicians	Family/Internal medicine	UK	36	42	0.857	[0.715; 0.946]	2.9%
Sahin 2023	Physicians	Family/Internal medicine	Turkey	148	156	0.949	[0.901; 0.978]	3.2%
Savoia 2023	Physicians	Family/Internal medicine	Europe	226	239	0.946	[0.909; 0.971]	3.4%
Waheed 2024	Physicians	Family/Internal medicine	Qatar	77	108	0.713	[0.618; 0.796]	3.6%
**Random effects model**					**1066**	**0.853**	**[0.675; 0.942]**	**16.9%**
**Prediction interval**							**[0.151; 0.995]**	
Heterogeneity: I^2 = 96.2%, τ^2 = 1.2997, χ^2_{4} = 104.04 (p < 0.001)								
**Speciatly = Emergency medicine**								
Eastwood 2023	Physicians	Emergency medicine	Canada	130	171	0.760	[0.689; 0.822]	3.7%
**Speciatly = Orthodontics**								
Mengi 2024	Phy+Stu	Orthodontics	India	96	100	0.960	[0.901; 0.989]	2.7%
**Speciatly = Radiology**								
Zanardo 2024	Physicians	Radiology	Europe	298	572	0.521	[0.479; 0.563]	3.8%
**Random effects model**					**13747**	**0.793**	**[0.739; 0.838]**	**100.0%**
**Prediction interval**							**[0.425; 0.952]**	

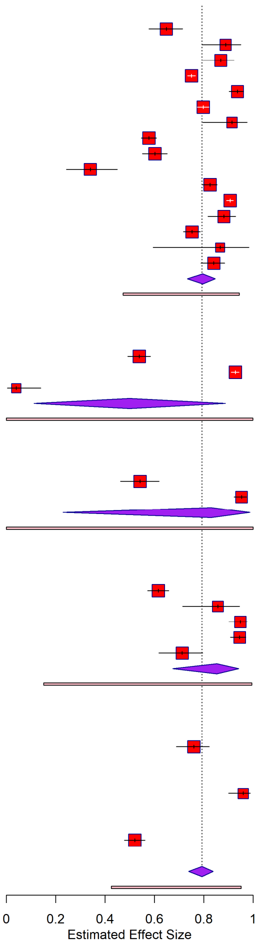

Heterogeneity: I^2 = 97.7%, τ^2 = 0.6196, χ^2_{28} = 1236.58 (p < 0.001)
Test for subgroup differences: χ^2_{6} = 95.76, df = 6 (p < 0.001)

Figure S12. Rates Of **Low or Medium AI Knowledge** Across the Included Studies, Stratified by the **Specialty** of the Participants. Each study's effect size represents the percentage of participants with low or medium AI knowledge in that study. The dotted line indicates the overall estimated rate of low or medium AI knowledge.

Study	Participants	Specialty	Country	Case N	Total N	Effect Size	95%CI	w.random
**Participant = Unknown**								
Al-Ali 2023	Unknown	Unknown	Saudi Arabia	41	72	0.569	[0.447; 0.686]	2.4%
Al-Medfa 2023	Unknown	Unknown	Bahrain	92	122	0.754	[0.668; 0.828]	2.4%
Al-Zaabi 2023	Unknown	Unknown	Oman	202	293	0.689	[0.633; 0.742]	2.5%
Bisdas 2021	Unknown	Unknown	International	2095	3033	0.691	[0.674; 0.707]	2.5%
Catalina 2023	Unknown	Unknown	Spain	258	301	0.857	[0.812; 0.895]	2.5%
Choi 2023	Unknown	Unknown	Korea	53	73	0.726	[0.609; 0.824]	2.4%
Civaner 2022	Unknown	Unknown	Turkey	181	3008	0.060	[0.052; 0.069]	2.5%
Eschert 2022	Unknown	Unknown	Germany	165	302	0.546	[0.488; 0.603]	2.5%
Gunasekeran 2022	Unknown	Unknown	International	865	1107	0.781	[0.756; 0.805]	2.5%
Hogberg 2023	Unknown	Unknown	Sweden	30	47	0.638	[0.485; 0.773]	2.3%
Huisman 2021	Unknown	Unknown	Europe	464	1039	0.447	[0.416; 0.477]	2.5%
Jussupow 2022	Unknown	Unknown	Unknown	104	230	0.452	[0.387; 0.519]	2.5%
Liu 2022	Unknown	Unknown	USA	54	388	0.139	[0.106; 0.178]	2.5%
Pecqueux 2022	Unknown	Unknown	Germany	84	144	0.583	[0.498; 0.665]	2.5%
Pinto 2019	Unknown	Unknown	Germany	137	263	0.521	[0.459; 0.583]	2.5%
Pucchio 2022	Unknown	Unknown	Canada	182	486	0.374	[0.331; 0.419]	2.5%
Scheetz 2021	Unknown	Unknown	Australia	457	632	0.723	[0.686; 0.758]	2.5%
Shiang 2022	Unknown	Unknown	Isreal	11	15	0.733	[0.449; 0.922]	1.9%
Valikodath 2021	Unknown	Unknown	USA	73	80	0.912	[0.828; 0.964]	2.2%
**Random effects model**					**11635**	**0.587**	**[0.449; 0.713]**	**46.0%**
**Prediction interval**							**[0.092; 0.952]**	
Heterogeneity: I^2 = 99.3%, τ^2 = 1.4947, χ^2_{18} = 2484.42 (p < 0.001)								
**Participant = Physicians**								
Al Medfa 2023	Physicians	Mixed	Bahrain	136	166	0.819	[0.752; 0.875]	2.4%
Alexandrou 2024	Physicians	Family/Internal medicine	North America + Europe	442	521	0.848	[0.815; 0.878]	2.5%
Cai 2024	Physicians	Anesthesiology	China	309	396	0.780	[0.736; 0.820]	2.5%
Eastwood 2023	Physicians	Emergency medicine	Canada	118	171	0.690	[0.615; 0.758]	2.5%
Eltorai 2024	Physicians	Radiology	USA	110	211	0.521	[0.452; 0.590]	2.5%
Estrada Alamo 2024	Physicians	Anesthesiology	USA	665	1083	0.614	[0.584; 0.643]	2.5%
Hantel 2024	Physicians	Family/Internal medicine	USA	111	203	0.547	[0.476; 0.617]	2.5%
Iyengar 2023	Physicians	Mixed	Europe + Asia	59	125	0.472	[0.382; 0.563]	2.5%
McGenity 2024	Physicians	Family/Internal medicine	UK	13	42	0.310	[0.176; 0.471]	2.3%
Nishan 2024	Physicians	Mixed	Bangladesh	283	398	0.711	[0.664; 0.755]	2.5%
Orlova 2023	Physicians	Mixed	Russia	150	301	0.498	[0.440; 0.556]	2.5%
Radhwi 2024	Physicians	Mixed	Saudi Arabia	52	128	0.406	[0.320; 0.497]	2.5%
Sahin 2023	Physicians	Family/Internal medicine	Turkey	55	156	0.353	[0.278; 0.433]	2.5%
Savoia 2023	Physicians	Family/Internal medicine	Europe	81	239	0.339	[0.279; 0.403]	2.5%
Waheed 2024	Physicians	Family/Internal medicine	Qatar	77	108	0.713	[0.618; 0.796]	2.4%
Zanardo 2024	Physicians	Radiology	Europe	419	572	0.733	[0.694; 0.768]	2.5%
**Random effects model**					**4820**	**0.600**	**[0.517; 0.678]**	**39.4%**
**Prediction interval**							**[0.256; 0.867]**	
Heterogeneity: I^2 = 96.4%, τ^2 = 0.4471, χ^2_{15} = 414.76 (p < 0.001)								
**Participant = Phy+Stu**								
Dumitrascu 2024	Phy+Stu	Unknown	Romania	247	309	0.799	[0.750; 0.843]	2.5%
Mengi 2024	Phy+Stu	Orthodontics	India	70	100	0.700	[0.600; 0.788]	2.4%
Tangadulrat 2023	Phy+Stu	Unknown	Thailand	188	239	0.787	[0.729; 0.837]	2.5%
**Random effects model**					**648**	**0.772**	**[0.718; 0.818]**	**7.4%**
**Prediction interval**							**[0.551; 0.903]**	
Heterogeneity: I^2 = 54.5%, τ^2 = 0.0342, χ^2_2 = 4.39 (p = 0.11)								
**Participant = Students**								
Lugito 2024	Students	Unknown	Indonesia	641	650	0.986	[0.974; 0.994]	2.3%
Malesivc 2024	Students	Unknown	East Europe	1308	1701	0.769	[0.748; 0.789]	2.5%
Robleto 2024	Students	Unknown	USA	32	72	0.444	[0.327; 0.566]	2.4%
**Random effects model**					**2423**	**0.849**	**[0.492; 0.970]**	**7.2%**
**Prediction interval**							**[0.003; 1.000]**	
Heterogeneity: I^2 = 98.3%, τ^2 = 2.3507, χ^2_2 = 119.41 (p < 0.001)								
**Random effects model**					**19526**	**0.628**	**[0.551; 0.700]**	**100.0%**
**Prediction interval**							**[0.171; 0.933]**	

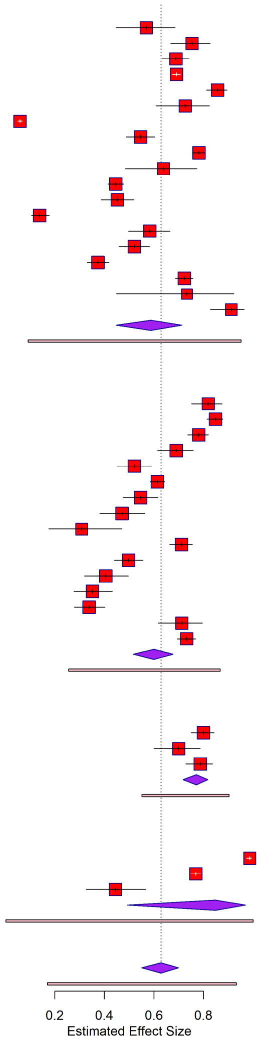

Heterogeneity: I^2 = 98.8%, τ^2 = 1.0600, χ^2_{40} = 3395.63 (p < 0.001)
Test for subgroup differences: χ^2_3 = 17.02, df = 3 (p < 0.001)

Figure S13. Rates Of **Medium or High AI Knowledge** Across the Included Studies, Stratified by the **Type** of the Participants. Each study's effect size represents the percentage of participants with medium or high AI knowledge in that study. The dotted line indicates the overall estimated rate of medium or high AI knowledge.

Study	Participants	Specialty	Country	Case N	Total N	Effect Size	95%CI	w.random
**Country = Asia**								
Al-Ali 2023	Unknown	Unknown	Saudi Arabia	41	72	0.569	[0.447; 0.686]	2.4%
Al-Medfa 2023	Unknown	Unknown	Bahrain	92	122	0.754	[0.668; 0.828]	2.4%
Al-Zaabi 2023	Unknown	Unknown	Oman	202	293	0.689	[0.633; 0.742]	2.5%
Al Medfa 2023	Physicians	Mixed	Bahrain	136	166	0.819	[0.752; 0.875]	2.4%
Cai 2024	Physicians	Anesthesiology	China	309	396	0.780	[0.736; 0.820]	2.5%
Choi 2023	Unknown	Unknown	Korea	53	73	0.726	[0.609; 0.824]	2.4%
Lugito 2024	Students	Unknown	Indonesia	641	650	0.986	[0.974; 0.994]	2.3%
Mengi 2024	Phy+Stu	Orthodontics	India	70	100	0.700	[0.600; 0.788]	2.4%
Nishan 2024	Physicians	Mixed	Bangladesh	283	398	0.711	[0.664; 0.755]	2.5%
Orlova 2023	Physicians	Mixed	Russia	150	301	0.498	[0.440; 0.556]	2.5%
Radhwi 2024	Physicians	Mixed	Saudi Arabia	52	128	0.406	[0.320; 0.497]	2.5%
Shiang 2022	Unknown	Unknown	Isreal	11	15	0.733	[0.449; 0.922]	1.9%
Tangadulrat 2023	Phy+Stu	Unknown	Thailand	188	239	0.787	[0.729; 0.837]	2.5%
Waheed 2024	Physicians	Family/Internal medicine	Qatar	77	108	0.713	[0.618; 0.796]	2.4%
**Random effects model**					**3061**	**0.737**	**[0.652; 0.806]**	**33.5%**
**Prediction interval**							**[0.355; 0.934]**	
Heterogeneity: $I^2 = 94.7\%$, $\tau^2 = 0.5258$, $\chi^2_{13} = 244.33$ ($p < 0.001$)								
**Country = International**								
Alexandrou 2024	Physicians	Family/Internal medicine	North America + Europe	442	521	0.848	[0.815; 0.878]	2.5%
Bisdas 2021	Unknown	Unknown	International	2095	3033	0.691	[0.674; 0.707]	2.5%
Gunasekeran 2022	Unknown	Unknown	International	865	1107	0.781	[0.756; 0.805]	2.5%
Iyengar 2023	Physicians	Mixed	Europe + Asia	59	125	0.472	[0.382; 0.563]	2.5%
**Random effects model**					**4786**	**0.719**	**[0.610; 0.807]**	**10.0%**
**Prediction interval**							**[0.308; 0.936]**	
Heterogeneity: $I^2 = 97.2\%$, $\tau^2 = 0.2387$, $\chi^2_3 = 108.55$ ($p < 0.001$)								
**Country = Europe**								
Catalina 2023	Unknown	Unknown	Spain	258	301	0.857	[0.812; 0.895]	2.5%
Civaner 2022	Unknown	Unknown	Turkey	181	3008	0.060	[0.052; 0.069]	2.5%
Dumitrascu 2024	Phy+Stu	Unknown	Romania	247	309	0.799	[0.750; 0.843]	2.5%
Eschert 2022	Unknown	Unknown	Germany	165	302	0.546	[0.488; 0.603]	2.5%
Hogberg 2023	Unknown	Unknown	Sweden	30	47	0.638	[0.485; 0.773]	2.3%
Huisman 2021	Unknown	Unknown	Europe	464	1039	0.447	[0.416; 0.477]	2.5%
Malesivc 2024	Students	Unknown	East Europe	1308	1701	0.769	[0.748; 0.789]	2.5%
McGenity 2024	Physicians	Family/Internal medicine	UK	13	42	0.310	[0.176; 0.471]	2.3%
Pecqueux 2022	Unknown	Unknown	Germany	84	144	0.583	[0.498; 0.665]	2.5%
Pinto 2019	Unknown	Unknown	Germany	137	263	0.521	[0.459; 0.583]	2.5%
Sahin 2023	Physicians	Family/Internal medicine	Turkey	55	156	0.353	[0.278; 0.433]	2.5%
Savoia 2023	Physicians	Family/Internal medicine	Europe	81	239	0.339	[0.279; 0.403]	2.5%
Zanardo 2024	Physicians	Radiology	Europe	419	572	0.733	[0.694; 0.768]	2.5%
**Random effects model**					**8123**	**0.529**	**[0.340; 0.711]**	**32.0%**
**Prediction interval**							**[0.043; 0.966]**	
Heterogeneity: $I^2 = 99.4\%$, $\tau^2 = 2.0334$, $\chi^2_{12} = 2110.83$ ($p < 0.001$)								
**Country = N America**								
Eastwood 2023	Physicians	Emergency medicine	Canada	118	171	0.690	[0.615; 0.758]	2.5%
Eltorai 2024	Physicians	Radiology	USA	110	211	0.521	[0.452; 0.590]	2.5%
Estrada Alamo 2024	Physicians	Anesthesiology	USA	665	1083	0.614	[0.584; 0.643]	2.5%
Hantel 2024	Physicians	Family/Internal medicine	USA	111	203	0.547	[0.476; 0.617]	2.5%
Liu 2022	Unknown	Unknown	USA	54	388	0.139	[0.106; 0.178]	2.5%
Pucchio 2022	Unknown	Unknown	Canada	182	486	0.374	[0.331; 0.419]	2.5%
Robleto 2024	Students	Unknown	USA	32	72	0.444	[0.327; 0.566]	2.4%
Valikodath 2021	Unknown	Unknown	USA	73	80	0.912	[0.828; 0.964]	2.2%
**Random effects model**					**2694**	**0.530**	**[0.386; 0.670]**	**19.5%**
**Prediction interval**							**[0.124; 0.900]**	
Heterogeneity: $I^2 = 97.7\%$, $\tau^2 = 0.6819$, $\chi^2_7 = 299.64$ ($p < 0.001$)								
**Country = Unknown**								
Jussupow 2022	Unknown	Unknown	Unknown	104	230	0.452	[0.387; 0.519]	2.5%
**Country = Pacific**								
Scheetz 2021	Unknown	Unknown	Australia	457	632	0.723	[0.686; 0.758]	2.5%
**Random effects model**					**19526**	**0.628**	**[0.551; 0.700]**	**100.0%**
**Prediction interval**							**[0.171; 0.933]**	

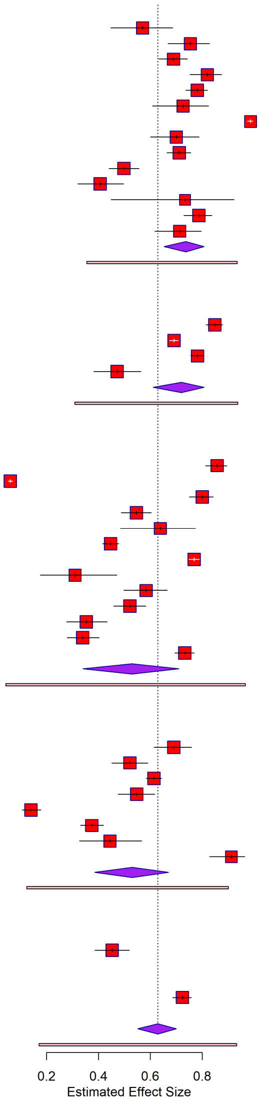

Heterogeneity: $I^2 = 98.8\%$, $\tau^2 = 1.0600$, $\chi^2_{40} = 3395.63$ ($p < 0.001$)
Test for subgroup differences: $\chi^2_5 = 62.15$, df = 5 ($p < 0.001$)

Figure S14. Rates Of **Medium or High AI Knowledge** Across the Included Studies, Stratified by the **Country** of the Participants. Each study's effect size represents the percentage of participants with medium or high AI knowledge in that study. The dotted line indicates the overall estimated rate of medium or high AI knowledge.

Study	Participants	Specialty	Country	Case N	Total N	Effect Size	95%CI	w.random
**Specialty = Unknown**								
Al-Ali 2023	Unknown	Unknown	Saudi Arabia	41	72	0.569	[0.447; 0.686]	2.4%
Al-Medfa 2023	Unknown	Unknown	Bahrain	92	122	0.754	[0.668; 0.828]	2.4%
Al-Zaabi 2023	Unknown	Unknown	Oman	202	293	0.689	[0.633; 0.742]	2.5%
Bisdas 2021	Unknown	Unknown	International	2095	3033	0.691	[0.674; 0.707]	2.5%
Catalina 2023	Unknown	Unknown	Spain	258	301	0.857	[0.812; 0.895]	2.5%
Choi 2023	Unknown	Unknown	Korea	53	73	0.726	[0.609; 0.824]	2.4%
Civaner 2022	Unknown	Unknown	Turkey	181	3008	0.060	[0.052; 0.069]	2.5%
Dumitrascu 2024	Phy+Stu	Unknown	Romania	247	309	0.799	[0.750; 0.843]	2.5%
Eschert 2022	Unknown	Unknown	Germany	165	302	0.546	[0.488; 0.603]	2.5%
Gunasekeran 2022	Unknown	Unknown	International	865	1107	0.781	[0.756; 0.805]	2.5%
Hogberg 2023	Unknown	Unknown	Sweden	30	47	0.638	[0.485; 0.773]	2.3%
Huisman 2021	Unknown	Unknown	Europe	464	1039	0.447	[0.416; 0.477]	2.5%
Jussupow 2022	Unknown	Unknown	Unknown	104	230	0.452	[0.387; 0.519]	2.5%
Liu 2022	Unknown	Unknown	USA	54	388	0.139	[0.106; 0.178]	2.5%
Lugito 2024	Students	Unknown	Indonesia	641	650	0.986	[0.974; 0.994]	2.3%
Malesivc 2024	Students	Unknown	East Europe	1308	1701	0.769	[0.748; 0.789]	2.5%
Pecqueux 2022	Unknown	Unknown	Germany	84	144	0.583	[0.498; 0.665]	2.5%
Pinto 2019	Unknown	Unknown	Germany	137	263	0.521	[0.459; 0.583]	2.5%
Pucchio 2022	Unknown	Unknown	Canada	182	486	0.374	[0.331; 0.419]	2.5%
Robleto 2024	Students	Unknown	USA	32	72	0.444	[0.327; 0.566]	2.4%
Scheetz 2021	Unknown	Unknown	Australia	457	632	0.723	[0.686; 0.758]	2.5%
Shiang 2022	Unknown	Unknown	Isreal	11	15	0.733	[0.449; 0.922]	1.9%
Tangadulrat 2023	Phy+Stu	Unknown	Thailand	188	239	0.787	[0.729; 0.837]	2.5%
Valikodath 2021	Unknown	Unknown	USA	73	80	0.912	[0.828; 0.964]	2.2%
**Random effects model**					**14606**	**0.647**	**[0.530; 0.748]**	**58.2%**
**Prediction interval**							**[0.129; 0.958]**	
Heterogeneity: I^2 = 99.2%, τ^2 = 1.4131, χ^2_{23} = 2965.72 (p < 0.001)								
**Specialty = Mixed**								
Al Medfa 2023	Physicians	Mixed	Bahrain	136	166	0.819	[0.752; 0.875]	2.4%
Iyengar 2023	Physicians	Mixed	Europe + Asia	59	125	0.472	[0.382; 0.563]	2.5%
Nishan 2024	Physicians	Mixed	Bangladesh	283	398	0.711	[0.664; 0.755]	2.5%
Orlova 2023	Physicians	Mixed	Russia	150	301	0.498	[0.440; 0.556]	2.5%
Radhwi 2024	Physicians	Mixed	Saudi Arabia	52	128	0.406	[0.320; 0.497]	2.5%
**Random effects model**					**1118**	**0.594**	**[0.441; 0.731]**	**12.3%**
**Prediction interval**							**[0.152; 0.923]**	
Heterogeneity: I^2 = 95.6%, τ^2 = 0.4722, χ^2_4 = 90.2 (p < 0.001)								
**Specialty = Family/Internal medicine**								
Alexandrou 2024	Physicians	Family/Internal medicine	North America + Europe	442	521	0.848	[0.815; 0.878]	2.5%
Hantel 2024	Physicians	Family/Internal medicine	USA	111	203	0.547	[0.476; 0.617]	2.5%
McGenity 2024	Physicians	Family/Internal medicine	UK	13	42	0.310	[0.176; 0.471]	2.3%
Sahin 2023	Physicians	Family/Internal medicine	Turkey	55	156	0.353	[0.278; 0.433]	2.5%
Savoia 2023	Physicians	Family/Internal medicine	Europe	81	239	0.339	[0.279; 0.403]	2.5%
Waheed 2024	Physicians	Family/Internal medicine	Qatar	77	108	0.713	[0.618; 0.796]	2.4%
**Random effects model**					**1269**	**0.533**	**[0.318; 0.737]**	**14.6%**
**Prediction interval**							**[0.050; 0.961]**	
Heterogeneity: I^2 = 97.9%, τ^2 = 1.2192, χ^2_5 = 234.45 (p < 0.001)								
**Specialty = Anesthesiology**								
Cai 2024	Physicians	Anesthesiology	China	309	396	0.780	[0.736; 0.820]	2.5%
Estrada Alamo 2024	Physicians	Anesthesiology	USA	665	1083	0.614	[0.584; 0.643]	2.5%
**Random effects model**					**1479**	**0.702**	**[0.518; 0.838]**	**5.0%**
**Prediction interval**							**[0.000; 1.000]**	
Heterogeneity: I^2 = 97.1%, τ^2 = 0.3132, χ^2_1 = 34.63 (p < 0.001)								
**Specialty = Emergency medicine**								
Eastwood 2023	Physicians	Emergency medicine	Canada	118	171	0.690	[0.615; 0.758]	2.5%
**Specialty = Radiology**								
Eltorai 2024	Physicians	Radiology	USA	110	211	0.521	[0.452; 0.590]	2.5%
Zanardo 2024	Physicians	Radiology	Europe	419	572	0.733	[0.694; 0.768]	2.5%
**Random effects model**					**783**	**0.635**	**[0.413; 0.811]**	**5.0%**
**Prediction interval**							**[0.000; 1.000]**	
Heterogeneity: I^2 = 96.7%, τ^2 = 0.4112, χ^2_1 = 30.46 (p < 0.001)								
**Specialty = Orthodontics**								
Mengi 2024	Phy+Stu	Orthodontics	India	70	100	0.700	[0.600; 0.788]	2.4%
**Random effects model**					**19526**	**0.628**	**[0.551; 0.700]**	**100.0%**
**Prediction interval**							**[0.171; 0.933]**	

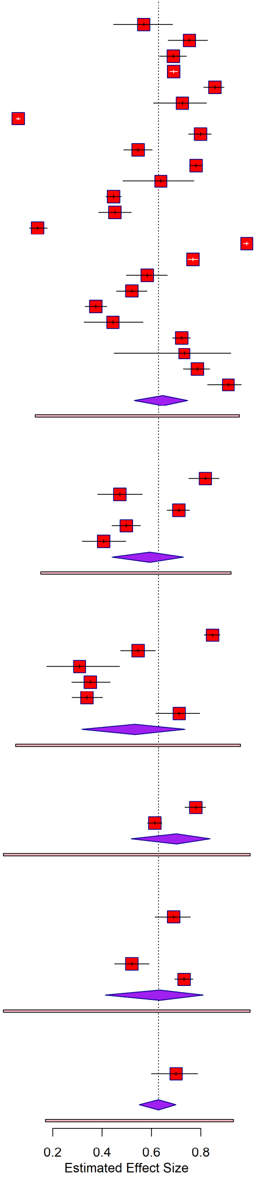

Heterogeneity: I^2 = 98.8%, τ^2 = 1.0600, χ^2_{40} = 3395.63 (p < 0.001)
Test for subgroup differences: χ^2_6 = 3.79, df = 6 (p = 0.70)

Figure S15. Rates Of **Medium or High AI Knowledge** Across the Included Studies, Stratified by the **Specialty** of the Participants. Each study's effect size represents the percentage of participants with medium or high AI knowledge in that study. The dotted line indicates the overall estimated rate of medium or high AI knowledge.

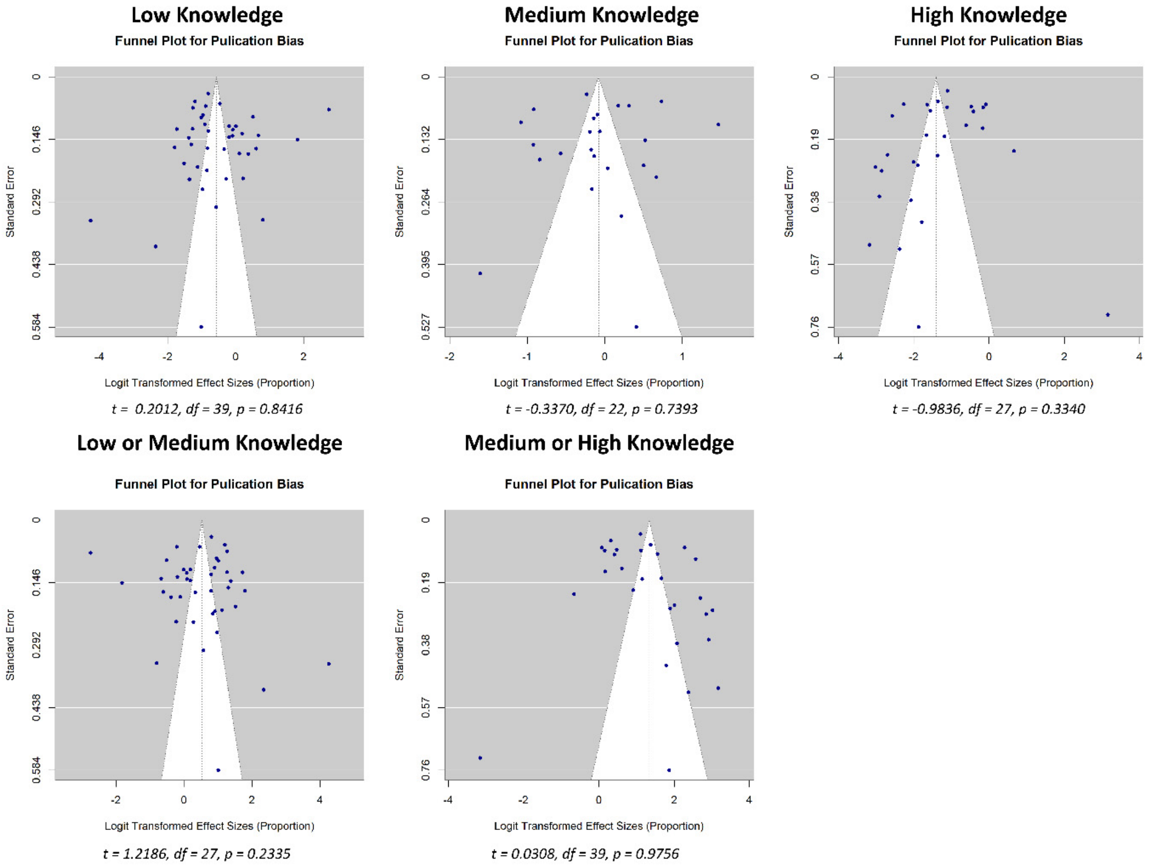

Figure S16. Funnel Plots Evaluating the Risk of Publication Bias Among the Included Studies. Results of Egger's tests are indicated below each funnel plot.
